# Assessing the impact of anaesthetic and surgical task-shifting globally: a systematic literature review

**DOI:** 10.1093/heapol/czad059

**Published:** 2023-07-28

**Authors:** Maeve S Bognini, Christian I Oko, Meskerem A Kebede, Martilord I Ifeanyichi, Darshita Singh, Rachel Hargest, Rocco Friebel

**Affiliations:** Global Surgery Policy Unit, The London School of Economics and Political Science, Houghton Street, London WC2A 2AE, UK; Division of Health Research, Lancaster University, Bailrigg, Lancaster LA1 4YW, United Kingdom; Global Surgery Policy Unit, The London School of Economics and Political Science, Houghton Street, London WC2A 2AE, UK; Global Surgery Policy Unit, The London School of Economics and Political Science, Houghton Street, London WC2A 2AE, UK; Global Surgery Policy Unit, The London School of Economics and Political Science, Houghton Street, London WC2A 2AE, UK; Global Surgery Policy Unit, The London School of Economics and Political Science, Houghton Street, London WC2A 2AE, UK; University Hospital of Wales, Heath Park, Cardiff CF14 4XN, United Kingdom; Global Surgery Policy Unit, The London School of Economics and Political Science, Houghton Street, London WC2A 2AE, UK; Center for Global Development, Abbey Gardens, Great College Street, London SW1P 3SE, United Kingdom

**Keywords:** Global surgery, anaesthesia workforce, surgical workforce, task-shifting, human resources for health, non-specialist physicians, non-physician clinicians, healthcare delivery

## Abstract

The global shortage of skilled anaesthesiologists, surgeons and obstetricians is a leading cause of high unmet surgical need. Although anaesthetic and surgical task-shifting are widely practised to mitigate this barrier, little is known about their safety and efficacy. This systematic review seeks to highlight the existing evidence on the clinical outcomes of patients operated on by non-physicians or non-specialist physicians globally. Relevant articles were identified by searching four databases (MEDLINE, EMBASE, CINAHL and Global Health) in all languages between January 2008 and February 2022. Retrieved documents were screened against pre-specified inclusion and exclusion criteria, and their qualities were appraised critically. Data were extracted by two independent reviewers and findings were synthesized narratively. In total, 40 studies have been included. Thirty-five focus on task-shifting for surgical and obstetric procedures, whereas four studies address anaesthetic task-shifting; one study covers both interventions. The majority are located in sub-Saharan Africa and the USA. Seventy-five per cent present perioperative mortality outcomes and 85% analyse morbidity measures. Evidence from low- and middle-income countries, which primarily concentrates on caesarean sections, hernia repairs and surgical male circumcisions, points to the overall safety of non-surgeons. On the other hand, the literature on surgical task-shifting in high-income countries (HICs) is limited to nine studies analysing tube thoracostomies, neurosurgical procedures, caesarean sections, male circumcisions and basal cell carcinoma excisions. Finally, only five studies pertaining to anaesthetic task-shifting across all country settings answer the research question with conflicting results, making it difficult to draw conclusions on the quality of non-physician anaesthetic care. Overall, it appears that non-specialists can safely perform high-volume, low-complexity operations. Further research is needed to understand the implications of surgical task-shifting in HICs and to better assess the performance of non-specialist anaesthesia providers. Future studies must adopt randomized study designs and include long-term outcome measures to generate high-quality evidence.

Key messages

- Although non-specialists are the main providers of surgical care in many resource-limited settings, the impact of anaesthetic and surgical task-shifting on patients’ clinical outcomes remains poorly understood.
- In low- and middle-income countries, non-specialists perform comparably to specialist providers of surgical care in low-complexity, high-volume procedures (caesarean sections, hernia repairs and surgical male circumcisions).
- Although surgical task-shifting is predominantly practised in low- and middle-income settings, non-physician clinicians and non-specialist physicians also operate in high-income countries to reduce the lack of specialist surgeons.
- Future research must further investigate the safety of anaesthetic task-shifting globally.

## Introduction

Evidence shows that there is a significant unmet ‘surgical need’ globally, with up to 5 billion people facing barriers to accessing surgical care and 2 billion people without any access (Funk *et al.*, 2010). It is estimated that between 11% and 32% of the global burden of disease is surgical in nature (Debas *et al.*, 2006), and >6% of all global avertable deaths and disabilities are preventable through the provision of essential surgery (Mock *et al.*, 2015). These figures are likely to worsen in the future due to the epidemiological shift towards non-communicable diseases and traumatic injuries and because of an ongoing maternal health crisis in low-resource settings (Luboga *et al.*, 2009; Hoyler *et al.*, 2014).

The shortage of trained workforce in anaesthesia and surgery is one of the primary barriers to patients receiving surgical care (Meara *et al.*, 2015). According to the Lancet Commission on Global Surgery, countries with <20 surgeons, anaesthesiologists and obstetricians per 100 000 population may experience a health workforce crisis although this target is rarely met in resource-poor settings (Hoyler *et al.*, 2014). Across 42 low- and middle-income countries (LMICs), national general surgeon density ranged between 0.13 and 1.57 per 100 000 population; obstetrician density was found to be between 0.042 and 12.5 per 100 000 population and anaesthesiologist density ranged between 0 and 4.9 per 100 000 population (Hoyler *et al.*, 2014). This challenge also increasingly affects high-income countries (HICs), where rural catchment areas are often too small to support specialist physicians (Kornelsen *et al.*, 2012; Grzybowski *et al.*, 2013; Homan *et al.*, 2013). Due to the paucity of education and training opportunities, combined with low pay and poor working conditions, anaesthesiologists and surgeons often opt to migrate internally, towards urban centres, or abroad. High rates of attrition lead to an inequitable distribution of the healthcare workforce, with rural and deprived areas experiencing significant gaps in surgical coverage (Hoyler *et al.*, 2014; Henry *et al.*, 2015; Falk *et al.*, 2020).

To address this issue, the international community has widely advocated for the need to make more effective use of the existing healthcare workforce through task-shifting (WHO 2008; Luboga *et al.*, 2009; Meara *et al.*, 2015). This refers to the delivery of surgical procedures by individuals with shorter training and fewer qualifications than the specialist physicians who would normally perform surgeries. Surgical tasks can be delegated to two categories of healthcare professionals: non-physician clinicians (NPCs) and generalist physicians (non-specialists) (Federspiel *et al.*, 2018; Falk *et al.*, 2020). Task-shifting has been implemented across countries of all income groups: a review and survey conducted by Federspiel *et al.* (2015) identified 30 countries employing surgical task-shifting and 108 countries relying on anaesthetic task-shifting.

Despite the widespread adoption of this strategy, the evidence around its efficacy and acceptability remains fragmented and controversial. Its supporters primarily contend that task-shifting can ensure timely access to surgical care by expanding the pool of surgical providers and the skill-mix of operating teams (Dawson *et al.*, 2014; Binda *et al.*, 2021; Beard *et al.*, 2022). For example, non-specialists perform 52.8% of all surgeries in Sierra Leone and 58.3% of procedures in Liberia (Bolkan *et al.*, 2016; Adde *et al.*, 2022). Other perceived advantages include its cost-effectiveness, given the lower training and remuneration costs (Kruk *et al.*, 2007; Hounton *et al.*, 2009; Beard *et al.*, 2022), as well as higher retention rates of non-specialist clinicians (Chu *et al.*, 2009). However, specialist physicians, patients and policymakers have questioned the quality of surgical care provided through task-shifting. They fear that this strategy could result in the creation of a two-tier healthcare system where patients who are operated on by specialists receive superior care compared with those treated by less specialized cadres (Ashengo *et al.*, 2017; van Heemskerken *et al.*, 2020).

The safety of anaesthetic and surgical care provided by non-specialists has been insufficiently evaluated, and it remains poorly understood (Federspiel *et al.*, 2018; Schroeder *et al.*, 2020). Our systematic review synthesizes the existing literature reporting on anaesthetic and surgical outcomes of patients who are operated on by non-physicians and non-specialist physicians globally. As insufficient capacity is one of the main barriers to delivering surgical care worldwide, it is important to investigate the feasibility and safety of task-shifting to mitigate the shortage of healthcare providers.

## Methods

We conducted a systematic literature review, in compliance with the Preferred Reporting Items for Systematic Reviews and Meta-Analyses (PRISMA) guidelines (Moher *et al.*, 2009). A study protocol was written and registered on PROSPERO prior to commencing the review process. On 22 February 2022, we searched four electronic databases for relevant literature: MEDLINE, EMBASE, CINAHL and Global Health. To identify other relevant publications, we manually searched the bibliographies of all included articles and the reference lists of existing reviews on surgical task-shifting.

We developed a comprehensive search strategy, in conjunction with a university librarian with expertise in Health Policy. This included combinations of keywords and subject headings relating to surgical disciplines (i.e. surgery, anaesthesia and obstetrics), task-shifting and synonyms (i.e. task-sharing and task-delegation) and non-specialist healthcare professionals (i.e. non-physician, non-specialist and non-surgeon). Although we generally selected broad search terms pertaining to anaesthesia and surgery, without focusing on a particular surgical discipline, we chose to include keywords for caesarean section, laparotomy and open fracture management. This decision was made as these Bellwether procedures are indicative of functioning and comprehensive district-level surgical care delivery systems (Meara *et al.*, 2015). The complete search strategy applied to MEDLINE can be viewed in Supplementary File 1.

### Inclusion and exclusion criteria

Our systematic review focused on studies published from January 2008 until February 2022. This search period was chosen based on the launch of the first global conference on task-shifting, organized by the World Health Organization (WHO) in 2008. The searches were not restricted by language or by country setting.

#### Population

Study participants were patients who received anaesthesia or sedation as part of a surgical procedure or who underwent surgery. A surgical procedure was defined as ‘the incision, excision or manipulation of tissue that requires regional or general anaesthesia or profound sedation to control pain’ (Meara *et al.*, 2015). Relevant procedures included both minor and major surgical operations, performed on urgent (emergency) or planned (elective) basis. Dental procedures and other medical services beyond anaesthesia and surgery were excluded. Patients of all ages and from all countries and healthcare settings were eligible for inclusion.

#### Intervention

Task-shifting was defined as the rational redistribution of anaesthetic and surgical tasks from highly qualified health workers to health workers with shorter training and fewer qualifications to maximize the available human resources for health (WHO, PEPFAR, UNAIDS, 2008). We focused on vertical task-shifting: anaesthesia and surgical tasks could be delegated from specialist surgeons or anaesthesiologists to non-specialist physicians or from physicians to NPCs. We included all studies in which non-specialists were the lead providers of anaesthetic or surgical care in the intervention arm. Given the feasibility in the scope of this work, it must be highlighted that we took a focused approach, concentrating on task-shifting in relation to the intraoperative performance of anaesthesia and surgery, rather than broader perioperative aspects of care (e.g. preoperative preparation or postoperative care) and non-technical skills (e.g. clinical decision-making).

#### Comparators

The comparator of interest was ‘the standard practice’: in the control group, anaesthesia and surgery were performed by the physicians who would ordinarily conduct the procedures in the study setting. Therefore, physicians could be specialists or non-specialists. A specialist was defined as a physician who had completed residency training in a surgical speciality or in anaesthesia (i.e. a qualified surgeon or anaesthesiologist). A non-specialist physician was as a medical doctor who had completed medical school but who did not have formal training in surgery or anaesthesia [i.e. general practitioner (GP)]. Studies without a relevant comparator were excluded.

#### Outcomes

Studies that reported on anaesthetic or surgical outcome data were included. The primary outcomes of this systematic review were short-term and long-term mortality measures. Secondary outcomes comprised all other patient clinical outcomes because of anaesthetic and surgical task-shifting. We anticipated that common secondary outcome measures would include morbidity measures, length of hospital stay, unplanned hospital readmission, reoperation and patient-reported outcomes.

#### Study designs

We included all randomized controlled trials (RCTs), quasi-experimental studies and prospective and retrospective observational studies and cross-sectional studies. Qualitative studies were excluded as the main clinical outcomes of interest centred around clinical safety, as opposed to patient perspectives and experiences of the anaesthetic and surgical care received. Existing literature reviews, case studies, conference abstracts and commentaries were not eligible.

### Study selection

After removing duplicates in EndNote, two authors independently screened all results against the pre-specified inclusion criteria to identify eligible studies: first, they reviewed ‘titles and abstracts’ and, subsequently, the full texts of the selected articles were reviewed. Discrepancies were resolved through discussions and by involving a third reviewer until a consensus was reached. The screening process was managed through Rayyan.

### Data extraction

The following data items were recorded for each study to establish combinability: the country setting and the healthcare facilities where patients were treated; the occurrence of either surgical or anaesthetic task-shifting and the characteristics of the procedures performed and the characteristics of surgical or anaesthetic providers, including the training received and the level of supervision of non-specialists. We also noted study designs and relevant clinical outcome variables.

In the event of missing data, we contacted study authors for clarification. All data points were independently extracted by one author into a pre-tested Microsoft Excel form; subsequently, a second author checked and confirmed the accuracy and completeness of the recorded information.

### Risk of bias

The risk of bias of randomized studies was appraised through Version 2 of the ‘Cochrane risk-of-bias tool for randomized trials’ (RoB 2) (Sterne *et al.*, 2019) (Tables 1 and 2). Domains included bias arising from the randomization process, bias due to deviations from the intended interventions, bias due to missing outcome data, bias in the measurement of outcomes and bias in the selection of reported results. Instead, we evaluated the risk of bias in non-randomized studies using the ‘Risk of bias in non-randomized studies of interventions’ instrument (ROBINS-I) (Sterne *et al.*, 2016). We assessed bias due to confounding, selection of participants into the study, classification of the interventions, deviations from the intended interventions, missing outcome data and measurement of outcomes.

**Table 1.** ROB-2, risk of bias judgements for randomized studies

Study name	Domain 1	Domain 2	Domain 3	Domain 4	Domain 5	Overall
Ashley *et al.*, 2021	Low	Low	Moderate	Low	Low	Moderate

**Table 2.** ROBINS-I, risk of bias judgements for non-randomized studies

Study names	Domain 1	Domain 2	Domain 3	Domain 4	Domain 5	Domain 6	Overall
Attebery *et al.*, 2010	Critical	Low	Serious	Low	Serious	Serious	Critical
Beard *et al.*, 2014	Moderate	Low	Low	Low	Serious	Moderate	Serious
Beard *et al.*, 2019	Moderate	Low	Low	Low	Moderate	Low	Moderate
Bevis *et al.*, 2008	Moderate	Moderate	Low	Moderate	Moderate	Low	Moderate
Bolkan *et al.*, 2017	Moderate	Low	Low	Low	Low	Low	Moderate
Buwembo *et al.*, 2011	Serious	Serious	Low	Low	Low	Moderate	Serious
Chu *et al.*, 2011	Critical	Low	Low	Low	Low	Low	Critical
Cometto *et al.*, 2012	Critical	Low	Low	Moderate	Low	Low	Critical
Dulisse and Cromwell, 2010	Moderate	Low	Low	Low	Low	Moderate	Moderate
Ellens *et al.*, 2019	Critical	Low	Low	Moderate	Low	Serious	Critical
Enriquez-Marulanda *et al.*, 2018	Critical	Moderate	Low	Moderate	Moderate	Moderate	Critical
Gajewski *et al.*, 2019a	Critical	Low	Low	Low	Low	Low	Critical
Gajewski, et al., 2019b	Critical	Low	Low	Low	Low	Low	Critical
Gerber *et al.*, 2019	Serious	Low	Low	Low	Low	Serious	Serious
Gessessew *et al.*, 2011	Critical	Low	Low	Low	Low	Low	Critical
Giramonti and Kogan, 2018	Critical	Serious	Low	Serious	Low	Low	Critical
Harrell *et al.*, 2020	Critical	Serious	Low	Low	Moderate	Moderate	Critical
Homan *et al.*, 2013	Critical	Low	Low	Low	Low	Moderate	Critical
Hounton *et al.*, 2009	Critical	Serious	Low	Low	Serious	Moderate	Critical
Kudsk-Iversen *et al.*, 2020	Moderate	Serious	Low	Serious	Serious	Low	Serious
Matinhure *et al.*, 2018	Serious	Moderate	Low	Low	Low	Moderate	Serious
Matumaini *et al.*, 2021	Moderate	Low	Low	Low	Low	Moderate	Moderate
McCord *et al.*, 2009	Moderate	Low	Low	Low	Low	Moderate	Moderate
Mpirimbanyi *et al.*, 2017	Critical	Low	Low	Low	Serious	Moderate	Critical
Needleman and Minnick, 2009	Moderate	Low	Low	Low	Low	Moderate	Moderate
Ngcobo *et al.*, 2018	Critical	Moderate	Low	Low	Low	Moderate	Critical
Ouédraogo *et al.*, 2015	Critical	Low	Low	Low	Serious	Moderate	Critical
Ramdas *et al.*, 2018	Moderate	Serious	Low	Low	Moderate	Serious	Serious
Rech *et al.*, 2014	Critical	Low	Low	Low	Low	Low	Critical
Tariku *et al.*, 2019	Moderate	Moderate	Low	Low	Serious	Low	Serious
Thevi *et al.*, 2017	Critical	Low	Serious	Moderate	Serious	Moderate	Critical
Tobome *et al.*, 2021	Critical	Low	Low	Low	Low	Moderate	Critical
Tyson *et al.*, 2014	Serious	Low	Low	Serious	Serious	Moderate	Serious
van der Merwe *et al.*, 2021	Moderate	Low	Low	Low	Low	Moderate	Moderate
Van Duinen *et al.*, 2019	Serious	Serious	Low	Low	Serious	Moderate	Serious
Wamalwa *et al.*, 2015	Critical	Low	Low	Low	Moderate	Serious	Critical
Wilhelm *et al.*, 2011	Serious	Low	Low	Serious	Serious	Moderate	Serious
Wilhelm *et al.*, 2017	Serious	Low	Low	Serious	Serious	Moderate	Serious
Young and Bowling, 2012	Moderate	Low	Low	Low	Low	Moderate	Moderate

### Data analysis

Given the heterogeneous nature of the interventions and outcomes assessed, we could not pool study data in a meta-analysis. Instead, we performed a narrative synthesis of results. To describe the findings, studies were grouped by task-shifting category (surgery or anaesthesia), location (LMICs or HICs) and surgical procedure type.

## Results

### Search and screening results

**Figure 1.**
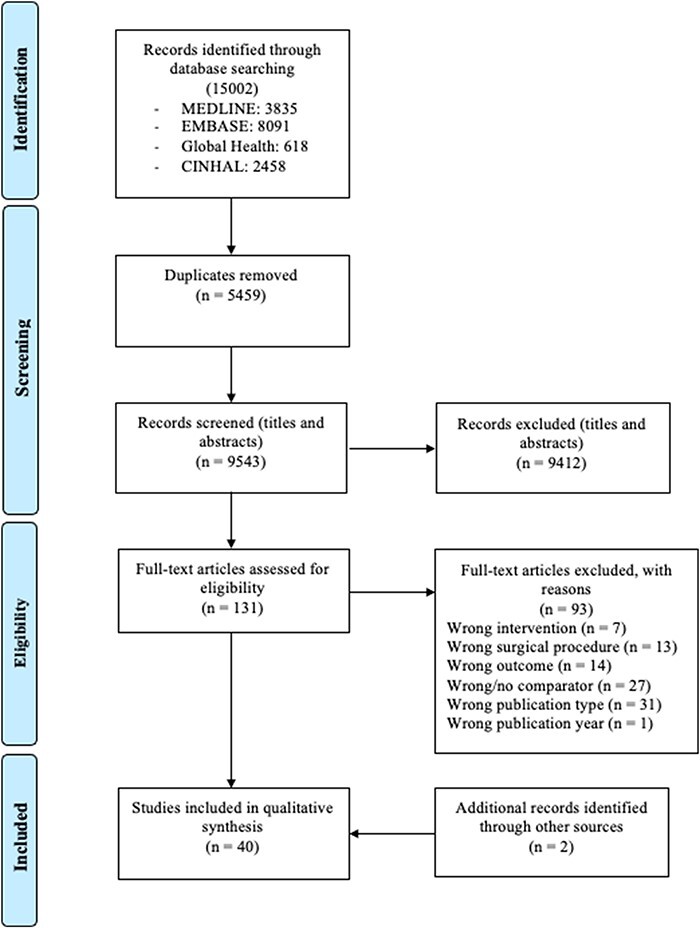
The PRISMA study flow diagram

Our initial search strategy identified 15 002 publications. Following duplicate removal, 9543 articles were screened at ‘title and abstract’ stage, and then, 131 full-text papers were reviewed. Two additional articles were identified through references, leading to a total of 40 studies being included in this systematic review. Further details are outlined in the PRISMA study flow diagram (Figure 1).

### Characteristics of the included studies

Thirty-five studies exclusively addressed surgical task-shifting (Table 3) and four focused on anaesthetic task-shifting (Table 4). One study analysed both interventions (Cometto *et al.*, 2012). Across all studies, the three most performed surgical procedures were caesarean sections (nine studies, 22.5%), male circumcisions (six studies, 15%) and hernia repairs (five studies, 12.5%). Thirty publications focused on delegation to NPCs, whereas in seven studies surgical or anaesthetic tasks were shifted to non-specialist physicians. In Chu *et al.* (2011) and Bolkan *et al.* (2017), the intervention group comprised both non-physicians and general medical doctors; Hounton *et al.* (2009) separately assessed the impact of task-shifting to both cadres.

**Table 3.** Characteristics of included studies, surgical task-shifting

Study name, country	Study design	Healthcare facility characteristics	Procedures	Lead surgical providers and number of procedures	Training and level of supervision of non-specialists	Outcomes assessed	Mortality	Other clinical outcomes
Ashley *et al.*, 2021. Sierra Leone (sub-Saharan Africa). Low-income economy.	Single-blind, parallel, non-inferiority randomized clinical trial. Data were collected between October 2017 and February 2019.	Single-centre study. First-level district hospital, serving an entirely rural catchment population of 200 000 people.	Surgical task-shifting. Anterior inguinal hernia repair with mesh. Major surgical procedure; elective.	Associate clinicians (ACs): non-physicians. ACs operated on 115 patients. Medical doctors (MDs): non-specialist physicians. MDs operated on 114 patients.	Background training: 3-year Diploma in Community Health and 2 years of work experience as Community Health Officers (CHOs). 3-year surgical training programme (including 1 year of internship), organized by CapaCare. ACs routinely performed hernia repair using tissue techniques as part of their jobs. Study training: inguinal hernia mesh repair training programme (1-day theoretical module + hands-on practice performing the surgeries under supervision, 1–3 days). Supervision: ACs in the study operated independently.	Primary outcomes: mortality and hernia recurrence (1 year after the operation). Secondary outcomes: postoperative complications (any, pain, impaired wound healing, wound infection, haematoma and reoperation) (2 weeks after surgery). Fewer groin symptoms, Inguinal Pain Questionnaire score, patient satisfaction and Self-Assessed Health Status (1 year after surgery).	No difference in mortality rates between ACs and MDs.	Hernia recurrence (1-year post-surgery): 0.9% (ACs) vs 6.9% (MDs), *P* < 0.001. ACs and MDs were equivalent for all other morbidity outcomes measured at 2 weeks and 1-year post-surgery.
Attebery *et al.*, 2010. Tanzania (sub-Saharan Africa). Lower-middle-income economy.	Retrospective audit review. Data were collected between January 2006 and September 2007.	Single-centre study. Rural referral hospital, serving 11 000 patients and vastly funded by the Norwegian Ministry of Health.	Surgical task-shifting. 51 patients underwent basic and emergency neurosurgical procedures (including burr hole surgery, craniotomy, skull fracture repair, ventriculo-peritoneal shunting (VP-shunting), myelomeningocele repair, laminectomy and exploratory biopsies).	Assistant medical officer (AMO): non-physician. The AMO operated on 38 patients. American neurosurgeon: specialist physician who either operated independently or alongside the AMO (task-sharing). 11 patients were operated on by the American neurosurgeon in conjunction with the AMO (task-sharing); 3 patients were operated on by the American neurosurgeon alone.	The non-physician was a qualified AMO. He routinely performed general surgery at Haydom Lutheran Hospital. Study training: a condensed training programme for neurosurgical procedures led by the American neurosurgeon. Level of supervision: in the intervention group, the AMO operated as the ‘lead surgeon’, but the degree of supervision received is unclear.	Mortality.	No significant difference in mortality rates between the AMO and the neurosurgeon.	
Beard *et al.*, 2014. Tanzania (sub-Saharan Africa). Lower-middle-income economy.	Retrospective records review. Data were collected in 2012.	Multi-centre study. 7 district, regional and mission hospitals.	Surgical task-shifting. Non-obstetric major surgical procedures and laparotomies for ruptured ectopic pregnancies. The five most common procedures were elective inguinal hernia repair, prostatectomy, exploratory laparotomy, hydrocoelectomy and emergency inguinal hernia repair. Major surgery; emergency and elective procedures.	Non-physician clinicians (NPCs): clinical officers (COs) or AMOs. NPCs performed 948 procedures. Physicians: 20 medical officers (MOs) (non-specialists) and 5 surgeons (specialists). Physicians performed 750 procedures.	Background training: COs were secondary school graduates who had completed 3 years of training and were qualified to practise medicine and perform minor surgeries. Although COs are not usually licenced to practise major surgery in Tanzania, they routinely performed major surgical procedures in one of the study sites. AMOs: 3 years of CO training + further 2-year training in common major obstetric and general surgical procedures. They are licenced to practise medicine and surgery in Tanzania. Supervision: NPCs operated independently.	Mortality (in-hospital). Morbidity (30 days postoperatively): all, wound infection; anaemia requiring blood transfusion; reoperation and readmission. Subgroup analysis: mortality and ‘all’ morbidity events were reported separately for the five most common surgical procedures (elective inguinal hernia repair, prostatectomy, exploratory laparotomy, hydrocelectomy and emergency inguinal hernia repair).	All major surgical procedures: no difference in mortality rates between NPCs and physicians. Subgroup analysis: no difference in mortality rates between NPCs and physicians for elective inguinal hernia repair, prostatectomy, exploratory laparotomy, hydrocelectomy and emergency inguinal hernia repair.	All major surgical procedures: NPCs and physicians were equivalent for all morbidity measures. Subgroup analysis: no difference in ‘all’ morbidity rates between NPCs and physicians for elective inguinal hernia repair, prostatectomy, exploratory laparotomy, hydrocelectomy and emergency inguinal hernia repair.
Beard *et al.*, 2019. Ghana (sub-Saharan Africa). Lower-middle-income economy.	Prospective cohort study with non-inferiority design. Data were collected between February 2017 and September 2018.	Single-centre study. Referral hospital under the management of the Ghana Health Service (public hospital).	Surgical task-shifting. Anterior tension-free mesh hernia repair, according to the Lichtenstein technique. Major surgical procedure. Elective surgery.	MDs: non-specialist physicians. MDs operated on 119 patients. General surgeons: specialist physicians. The general surgeons operated on 123 patients.	Background training: medical school and a 2-year general internship. MDs did not have formal training in surgery. Study training: 2-week theoretical course by the Ghana Hernia Society followed by hands-on practical training, performing hernia repairs with mesh under the supervision of consultant surgeons. The performance of MDs was evaluated by two trainers to determine their competence. Level of supervision: upon completion of the training programme, MDs were qualified to perform hernia repairs independently.	Primary outcomes: mortality and hernia recurrence (1-year post-surgery). Secondary outcomes: postoperative complications (any, impaired wound healing, superficial infection, haematoma/seroma, severe pain, other complications and intervention for complications) (2 weeks post-surgery). Fewer symptoms, Inguinal Pain Questionnaire score, patient satisfaction, and Self-Assessed Health Status score (1-year post-surgery).	No difference in mortality rates between MDs and general surgeons.	Hernia recurrence (1-year post-surgery): 0.9% (MDs) vs 2.8% (general surgeons), *P* < 0.001. No difference between MDs and general surgeons for all other morbidity outcomes measured 2 weeks and 1-year post-surgery.
Bevis *et al.*, 2008. Kansas (USA). High-income economy.	Single-centre study. Level I trauma hospital, intensive care unit or operating room. Data were collected between June 2003 and December 2003.	Single-centre study. Level I trauma hospital (intensive care unit or operating room).	Surgical task-shifting. 71 tube thoracostomies, performed on 51 patients. Major, emergency procedure.	Advanced practice providers (APPs): non-physicians. APPs performed 38 tube thoracostomy insertions on 24 patients. Trauma surgeons: specialist physicians. Trauma surgeons performed 33 tube thoracostomy insertions on 27 patients.	APPs were trained to perform tube thoracostomies through courses led by attending surgeons. Each APP performed 10 tube thoracostomy placements under direct supervision to establish competency. Upon completion of the training, APPs were qualified and licenced to perform tube thoracostomy placements under indirect supervision in Kansas.	Death as a result of the tube thoracostomy procedure and hospital length of stay. Incorrect tube placement: tube kinking, lateral drainage port extrapleural, tube extending caudad from the insertion site, abutment to mediastinum, intrafissure placement, intra-abdominal or transdiaphragmatic placement. Insertion complications: bleeding, re-expansion pulmonary oedema, loss of pulse and vasovagal phenomena. Outcome complications: dislodgement of chest tube, empyema, video-assisted thoracotomy or thoracoscopic surgery, need for chest tube reinsertion and reinsertion <5 hours after removal.	No death occurred as a direct result of tube thoracostomies.	Tube extending caudad from insertion site: 2.6% (APPs) vs 21% (surgeons), *P* = 0.02. No other significant differences in incorrect tube placement parameters. No insertion complications occurred during the study period and there were no differences in outcome complications between APPs and surgeons. The duration of hospital length of stay was equivalent between APPs and surgeons.
Bolkan *et al.*, 2017. Sierra Leone (sub-Saharan Africa). Low-income economy.	Prospective observational study. Data were collected between January 2011 and July 2016.	Multi-centre study. 16 partner hospitals, comprising district hospitals run by private not-for-profit organizations and government district hospitals. All hospitals had the adequate surgical capacity and 24-hour availability of MDs to perform surgery.	Surgical task-shifting. Surgical and obstetrical procedures. Procedures included caesarean section, hernia repair, laparotomy, appendicectomy, dilatation and curettage and hysterectomy. Minor and major surgeries; elective and emergency operations.	Trainees of the Capacare Surgical Training Programme and Surgical Assistant Community Health Officers (SACHOs). Trainees were CHOs (non-physicians) and junior MDs (non-specialists). SACHOs were CHOs who had graduated from the Capacare Surgical Training Programme (non-physicians). Trainees performed 4715 procedures under indirect supervision; SACHOs performed 2369 procedures. Trainers and supervisors were qualified MDs (non-specialists) and specialist surgeons or obstetricians. During the training period, supervisors performed 4515 operations. Following the training, supervisors performed 114 procedures.	Background training: CHOs have a 3-year basic medical diploma training and 2 years of postgraduate clinical practice. Junior MDs have to complete medical school and an internship to be eligible for the CapaCare Surgical Training Programm. CapaCare surgical training: 2-year training course. Introductory 6-month course (theoretical and practical training); three 6-month surgical rotations in district hospitals, engaging in all aspects of care of surgical patients. Trainees undergo refresher training at Masanga hospital. CHOs have to complete a further 1-year internship **i**n a tertiary surgical and maternal hospital, before being appointed as SACHOs. Supervision: All trainees and SACHOs are indirectly supervised by an unscrubbed MD when performing surgical and obstetrical procedures.	Crude in-hospital mortality. Mortality rates following trainees’ and SACHOs’ indirectly supervised operations are compared with the mortality rates of ‘observed operations’ (performed by trainers and supervisors).	Trainees: no significant differences in mortality rates between trainees and trainers. SACHOS: no significant differences in mortality rates between SACHOs and physicians.	
Buwembo *et al.*, 2011. Uganda (sub-Saharan Africa). Low-income economy.	Programme evaluation (prospective study). Data were collected between May 2006 and May 2010.	Healthcare clinics.	Surgical task-shifting. 5152 male surgical circumcisions, using the dorsal slit or sleeve surgical resection methods. Minor surgical procedure; elective surgery.	COs: non-physicians. COs performed 3218 male circumcisions. Trained and certified general physicians: non-specialists. Physicians performed 1934 male circumcisions.	During the study, COs were trained by a consultant urologist to perform male circumcisions. No details of the background training of COs were specified. COs operated independently.	Safety: moderate or severe adverse events related to the surgery and wound healing. Moderate adverse events were those requiring medical treatment; severe adverse events required surgical intervention. Outcomes were measured 24–48 hours post-surgery, 7–9 days post-surgery and 4 weeks post-surgery. Efficiency: operative time from first skin incision to wound closure and dressing.		Physicians experienced a higher rate of adverse events than COs (1.5% vs 0.6%, *P* = 0.007). There was no overall difference in operative duration between physicians and COs.
Chu *et al.*, 2011. Somalia (sub-Saharan Africa). Low-income economy.	Prospective study. Data were collected between October 2006 and December 2009.	Single-centre study. A private hospital supported by Médecins Sans Frontièrs (MSF).	Surgical task-shifting. 2086 surgical interventions, performed on 1602 patients. Elective and emergent procedures (70% of interventions were emergent); minor and major surgeries. 1591 interventions were trauma related.	Doctor with surgical skills (non-specialist physician) and surgical nurse (non-physician). They operated on patients between January 2008 and December 2009: the doctor performed 1119 procedures and the surgical nurse performed 314 procedures. The doctor performed 89% of elective cases; the surgical nurse performed 46% of all caesarean sections. Expatriate surgeons from MSF: specialist physicians. They operated on patients between October 2006 and January 2008. They performed 653 surgical operations.	Training of generalist MD: Background: the generalist doctor was ‘extremely competent, especially in trauma surgery’. He had gained surgical skills with the International Committee of the Red Cross and International Medical Corps and he had attended several training seminars. Study training: 2 years of training under MSF expatriate surgeons. The MSF programme included technical training in surgical and anaesthesia skills. Surgical nurse: Background training: the nurse had acquired surgical skills through informal, on-the-job training.	Operative mortality.	0.2% (non-specialists) vs 1.7% (expatriate surgeons), *P* < 0.001.	Study training: surgical and anaesthesia skills acquired through the MSF training programme prior to January 2008. Level of supervision: the generalist doctor and the surgical nurse operated without supervision between 2008 and 2009.
Cometto *et al.*, 2012. South Sudan (sub-Saharan Africa). Low-income economy.	Retrospective review. Data were collected between 2005 and 2010.	Multi-centre study. The programme was implemented in Primary Healthcare Centres, supported by the Italian non-governmental organisation (NGO) Comitato Collaborazione Medica.	Surgical task-shifting. 1543 patients underwent an operation during the surgical missions of Comitato Collaborazione Medica. Minor and major surgical procedures. Surgical procedures included hernia repairs (42%); skin, abscesses, lipomas and burns (17.76%); proctology (8.49%); Ob/Gyn and breast surgery (8.43%); thyroid and neck surgery (5.18%); urology (5.12%); digestive tract surgery (5.06%) and others (7.97%). The majority were elective procedures.	Surgical technicians: non-physicians. They performed 216 surgical operations. Visiting expatriate consultant surgeons: specialist physicians. They performed 1327 surgical operations.	Training: surgical technicians had a level of background training between nurses and physicians. They were trained through the Comitato Collaborazione Medica training programme and through the War Wounded Referral Hospital (Kenya) managed by the International Committee of the Red Cross. They developed competencies in perioperative surgical care and in performing minor and major surgery. Level of supervision: surgical technicians were supervised by surgeons when performing caesarean sections and hernia repairs.	Mortality.	No differences between surgical technicians and expatriate surgeons.	
Ellens *et al.*, 2019. Michigan (USA). High-income economy.	Retrospective cohort study. Data were collected between January 2012 and September 2016.	Single-centre study. Level I trauma centre, intensive care unit or emergency department.	Surgical task-shifting. External ventricular drain (EVD) placement, taking place in the intensive care unit or in the emergency department.	Mid-level practitioners (MLPs): non-physicians. MLPs first-attempted EVD placement in 238 patients. Neurosurgeons: specialist physicians. Neurosurgeons first-attempted EVD placement in 70 patients.	Training and supervision: MLPs received on-the-job training to perform EVD placement. They had to place at least five EVDs under the direct supervision of senior MLPs or experienced neurosurgeons prior to operating independently. MLPs were allowed to operate independently solely after the authorization of a neurosurgeon.	Glasgow Coma Scale score (pre-procedure and post-procedure), number of passes of the catheter per procedure, the accuracy of placement and complications (all haemorrhages, intraventricular haemorrhage, intraparenchymal haemorrhage, subdural haemorrhage and subarachnoid haemorrhage, infection and cerebrospinal fluid leak).		No differences between MLPs and neurosurgeons in placement accuracy, Glasgow Coma Scale scores and complication rates. 18 patients operated on by MLPs required multiple passes; in 14 cases, MLPs abandoned the procedure after three failed passes.
Enriquez-Marulanda *et al.*, 2018. Massachusetts (USA). High-income economy.	Retrospective cohort comparative study. Data were collected between June 2007 and June 2017.	Single-centre study. Neurosurgical service at an academic and teaching hospital.	Surgical task-shifting. 203 patients underwent EVD placement in patients with acute subarachnoid haemorrhage. Emergency procedures (91.2%). Major surgery.	Advanced practitioners [nurse practitioners (NPs) and physician assistants]: non-physicians. Advanced practitioners performed 87 (36.5%) EVD placements. Neurosurgeons or subspeciality clinical fellows: specialist physicians. They performed 151 (63.5%) EVD placements.	Training and supervision: advanced practitioners had to perform five successful EVD placements with little or no assistance in order to qualify to operate unsupervised. Attending neurosurgeon backup was available when intraprocedural complications occurred or if three failed attempts occurred without obtaining cerebrovascular fluid drainage.	Primary: EVD tract haemorrhages and accuracy of catheter tip placement. Secondary: number of catheter placement attempts, intraprocedural complications, EVD infection, EVD obstruction/non-functional EVD, catheter dislodgement, need for repositioning and replacement and duration of EVD catheter.		EVD obstruction or non-functional EVD: 21.8% (advanced practitioners) vs 11.9% (neurosurgeons), *P* = 0.04. No differences in primary outcomes between provider types. No significant differences in the number of catheter placement attempts, intraprocedural complications, EVD infections, catheter dislodgement, need for repositioning and replacement and duration of EVD catheter.
Gajewski *et al.*, 2019a. Malawi (sub-Saharan Africa) Low-income economy.	Prospective, cross-sectional comparison study.	Multi-centre study. 18 Government district hospitals with the capacity to deliver major surgery.	Surgical task-shifting. Hernia repairs, performed on 559 patients. Major surgery; elective and emergency procedures.	COs: non-physicians. COs performed 523 hernia repairs. MDs: non-specialist physicians. MDs performed 36 hernia repairs.	Background training: Diploma in Clinical Medicine and 2-year (minimum) work experience providing clinical and surgical care in district hospitals/rural healthcare facilities. Study training: COST-Africa training programme, leading to the accreditation of a B.Sc. in General Surgery by the University of Malawi College of Medicine. The programme included 4 months of theoretical training; a 24-month surgical placement in district hospitals; 8-month further training in advanced surgical skills.	Perioperative mortality and wound infection rates (in-hospital).	No surgical deaths occurred during the study period.	No differences between COs and MDs in wound infection rates.
Gajewski *et al.*, 2019b. Zambia (sub-Saharan Africa) Low-income economy.	Prospective, cross-sectional comparison study. The length of the intervention varied across hospitals between 8 and 24 months, with an average of 17-month duration.	Multi-centre study. 8 (public or mission) district hospitals with a functioning operating theatre and capacity to scale up surgery.	Surgical task-shifting. Caesarean sections and index general surgery procedures (comprising hysterectomy, salpingectomy, laparotomy, hernia and hydrocele repair). Major surgery.	Medical licentiates (MLs): non-physicians. MLs performed 725 procedures: 544 caesarean sections and 181 other index procedures. MDs: non-specialist physicians. They were the highest qualified cadre operating in the study hospitals. MDs performed 1002 operations: 770 caesarean sections and 232 other index procedures.	Background training: 2-year Advanced Diploma course that comprised a theoretical and a practical component (rotations in internal medicine, paediatrics, surgery and obstetrics and gynaecology) + 1-year internship. Study training: COST-Africa training programme. This included a 3-month course in surgery and a placement in district hospitals. Level of supervision: MLs who were deployed to the intervention district hospitals received quarterly supervision by specialist surgeons.	Mortality and wound infection rates for caesarean sections and index general surgery procedures.	1 surgical death following an exploratory laparotomy performed by an MD. No deaths were registered following caesarean sections.	Equivalent wound infection rates between MLs and MDs for caesarean sections and index general surgery procedures.
Gerber *et al.*, 2019. Texas (USA). High-income economy.	Retrospective patient chart review. Data were collected between 2012 and 2016.	Single-centre study. Texas Children’s Hospital, Houston.	Surgical task-shifting. 551 newborn circumcisions using the Gomco clamp. Minor surgical procedure.	APPs: non-physicians. APPs included physician assistants and NPs. APPs performed 314 circumcisions. Attending paediatric urologists: specialist physicians. Paediatric urologists performed 237 circumcisions.	Training and supervision: APPs underwent training led by attending urologists. Initially, they observed 10 newborn circumcisions; then, they first assisted the urologist on 10 procedures and they performed 10 circumcisions under direct supervision. Upon completion of the training, APPs were qualified to perform newborn circumcisions autonomously, with only indirect supervision from the attending surgeon.	Complications, 30-day return to the operating room, revision of circumcision, circumcision-related penile surgery and intraoperative bleeding.		No difference in complications, 30-day return to the operating room, revision of circumcision, circumcision-related penile surgery and intraoperative bleeding.
Gessessew *et al.*, 2011. Ethiopia (sub-Saharan Africa). Low-income economy.	Retrospective cohort study. Data were collected between January 2006 and December 2008.	Multi-centre study. 11 district hospitals and 2 health centres with CEmOC status.	Surgical task-shifting. Caesarean sections. Major, emergency and elective operations.	Health officers: non-physicians. Health officers performed 1574 caesarean sections, including 55.9% of emergency operations. Obstetricians: specialist physicians. Obstetricians performed 1261 caesarean sections, including 63.9% of elective operations.	Training and supervision: 3 years of training in public health and clinical medicine and 6–9 months of experience in CEmOC services (including obstetric surgery). Health officers operated independently.	Maternal and foetal deaths; hospital length of stay.	No difference in maternal and foetal death rates.	Equivalent duration of postoperative hospital length of stay.
Giramonti and Kogan, 2018. New York (USA). High-income economy.	Prospective study. Data were collected over 14 months.	Single-centre study. Albany Medical Center, Division of Urology.	Surgical task-shifting. 150 paediatric sleeve circumcisions using surgical loupes. Minor surgery; elective procedure.	NP: non-physician. The NP performed 100 operations. Urologist: specialist physician. The urologist performed 50 operations.	Background training: The NP had 15 years of experience in urology and over 5 years of observation of circumcision, hypospadias repair and hernia repair. She had performed over 60 office circumcisions for newborns using the Gomco clamp. Study training and supervision: The NP was first trained as an assistant to the attending surgeon in the operating room. Once sufficient knowledge was established, the NP performed 10 operations under direct supervision and 14 operations under indirect supervision. She then operated independently.	Operative time.		Equivalent operative time between the NP and the urologist.
Harrell *et al.*, 2020. Tennessee (USA). High-income economy.	Retrospective records review. Data were collected between January 2014 and January 2019.	Single-centre study. Hospital-based aeromedical service affiliated with a Level 1 trauma centre.	Surgical task-shifting. Tube thoracostomies. Major emergency procedures.	Aeromedical personnel (registered nurses and flight paramedics): non-physicians. They performed 49 prehospital tube thoracostomies (PTT). Physicians performed 98 hospital tube thoracostomies (HTT).	Training and supervision: Didactic training sessions to develop clinical reasoning, simulations using a manikin and in-hospital clinical experience assisting the trauma team performing the tube thoracostomy. Biannual competency checks in chest tube insertion. Qualified aeromedical personnel operated independently.	Mortality and complications (malposition, organ injury, dislodgement of chest tube and empyema and pneumonia). Time to chest tube placement, needle decompression, unilateral placement, initial drainage, small bore chest tube and chest tube days. Hospital length of stay, intensive care unit length of stay, ventilator days, emergency department disposition (ward floor, intensive care unit and operating theatre) and hospital disposition (home, rehabilitation and skilled nursing facility).	There were no differences in mortality rates between aeromedical personnel in the PTT and physicians in the HTT.	Time to chest tube placement: 21.92 minutes (PTT) vs 157.04 minutes (HTT), *P* < 0.001. Needle decompression: 41.7% (PTT) vs 4.1% (HTT), *P* < 0.001. Small bore chest tube: 0% (PTT) vs 9.2% (HTT), *P* = 0.056. Disposition to intensive care unit: 67.3% (PTT) and 34% (HTT), *P* = 0.006. Intensive care unit length of stay: 10.35 days (PTT) and 6.70 days (HTT), *P* = 0.034.
Homan *et al.*, 2013. New England (USA). High-income economy.	Retrospective chart review. Data were collected for the most recent 125 caesarean section deliveries at each hospital.	Multi-centre study. Two rural hospitals: one family medicine hospital (FMH) was staffed with family doctors performing caesarean sections ; in the second obstetric hospital (OBH), obstetricians performed caesarean sections.	Surgical task-shifting. 250 caesarean sections. Major surgical operations. Both elective and emergency operations.	Family physicians operating at the FMH: non-specialist physicians. They performed 125 caesarean sections over a period of 60 months. Obstetricians operating at the OBH: specialist physicians. They performed 125 caesarean sections over a period of 30 months.	Background training: one family physician was trained in caesarean deliveries during residency; one family physician completed a rural obstetrics fellowship programme; the third family physician was trained whilst employed in the National Health Service Corps in Alaska. All family physicians had performed 37–50 primary caesarean deliveries and assisted on 75–110 caesarean sections before being credentialed at the FMH. Level of supervision: family physicians were the primary providers of caesarean sections at the FMH during the study period.	Maternal outcomes: mortality, intraoperative complications, infectious complications, postoperative complications and maternal hospital length of stay. Neonatal outcomes: foetal death, gestational age, Apgar score, transfer to neonatal intensive care unit, readmission and hospital length of stay. Quality indicators: decision to incision time, total surgical time and surgery scheduled at ≥39 weeks.	No maternal deaths occurred during the study period. One foetal death at 38 weeks occurred at the FMH because of amniotic band syndrome with a cord accident (*P* = 1.0).	Total postoperative complications (mean): 0.03 (FMH) vs 0.12 (OBH), *P* = 0.03). Maternal hospital length of stay: 3.0 days (FMH) vs 2.6 days (OBH), *P* < 0.01). Total surgical time: 55.2 minutes vs 42.5 minutes, *P* < 0.01. No differences between FMH and OBH for maternal intraoperative complications and infectious complications. Neonatal outcomes: No differences between FMH and OBH. No differences between FMH and OBH in decision to incision time and surgery scheduled at ≥39 weeks.
Hounton *et al.*, 2009. Burkina Faso (sub-Saharan Africa). Low-income economy.	Retrospective, cross-sectional, facility-based survey. Data were collected for 2004 and 2005.	Multi-centre study. 22 public-sector facilities providing caesarean sections in 6 regions of Burkina Faso. These included national, regional and district hospitals. District hospitals comprised both rural and urban hospitals.	Surgical task-shifting. 2305 caesarean sections. Major surgical procedures; emergency operations.	COs: non-physicians. COs performed 733 caesarean sections. General doctors trained in essential surgery: non-specialist physicians. They performed 552 caesarean sections. Obstetricians: specialist physicians. They performed 1020 caesarean sections.	Background training of COs: COs were registered nurses with 2 additional years of training in surgery. Background training of general doctors: 6-month training programme in essential surgery.	Maternal death, perinatal death, postoperative complications (wound infection, wound dehiscence and haemorrhage), duration of caesarean sections and postoperative hospital length of stay.	Newborn case fatality rates: 198/1000 (COs) vs 125/1000 (general doctors) vs 99/1000 (obstetricians) (significant). No differences between provider types in maternal mortality rates.	Hospital length of stay: 9 days (COs) vs 9 days (general doctors) vs 6 days (obstetricians) (significant). Operative duration: 53 minutes (COs) vs 57 minutes (general doctors) vs 46 minutes (obstetricians) (significant). No differences between COs, general doctors and obstetricians in postoperative complications.
Matinhure *et al.*, 2018. Tanzania (sub-Saharan Africa). Lower-middle-income economy.	Retrospective, cross-sectional, documentary review. Data were collected between January 2014 and December 2015.	Multi-centre study. 3 primary health facilities (health centres) and 3 secondary health facilities (district hospitals).	Surgical task-shifting. 4302 caesarean sections. Major, elective and emergency operations.	AMOs: non-physicians. In total, AMOs performed 3544 caesarean sections. 88% were emergency caesarean sections and 12% were elective. MDs. In total, MDs performed 758 caesarean sections. 95% were emergency caesarean sections and 5% were elective.	Background training: AMOs were qualified COs with >3 years of experience. They had undergone a further 2 years of training (Advanced Diploma in Clinical Medicine), including rotations in obstetrics and gynaecology, surgery, child health and community medicine.	Early maternal outcomes (48 hours post-surgery): maternal death rate and complications. Neonatal outcomes (24 hours post-surgery): neonatal death rate and neonatal complications.	No significant differences between AMOs and MDs in maternal and neonatal mortality rates.	No significant differences between AMOs and MDs in maternal and neonatal complications.
Matumaini *et al.*, 2021. Uganda (sub-Saharan Africa). Low-income economy.	Prospective, observational study. Data were collected between September 2013 and February 2014.	Multi-centre study. 2 private, not-for-profit hospitals and 1 government public health facility.	Surgical task-shifting. 274 voluntary medical male circumcisions (VMMCs). Outpatient procedures, offered on a walk-in basis.	COs and nurses: non-physicians. In total, they performed 221 (80.7%) VMMCs. Physicians. In total, they performed 53 (19.3%) VMMCs.	Training: the background training of non-physicians was not described. Level of supervision: non-physicians operated independently.	Adverse events: any, post-surgical bleeding (2 hours post-surgery), excessive skin removal, fever post-surgery, pus discharge post-surgery and urethral injury (recorded 2 hours, 24 hours, 3 days and 7 days post-surgery). Operative time for circumcision.		Operative time: 15.45 minutes (non-physicians) vs 32.72 minutes (doctors), *P* < 0.001. No significant difference in the occurrence of adverse events between non-physicians and doctors.
McCord *et al.*, 2009. Tanzania (sub-Saharan Africa). Lower-middle-income economy.	Retrospective cohort study. Data were collected between January and May 2006.	Multi-centre study. 9 government district hospitals and 5 mission hospitals.	Surgical task-shifting. 1088 emergency major obstetrical surgeries. 778 operations were performed in government hospitals; 309 operations were performed in mission hospitals.	AMOs: non-physicians. AMOs operated on 945 patients. MOs: non-specialist physicians who were licenced to practise medicine and surgery. MOs worked independently or alongside an AMO (task-sharing). MOs operated on 143 patients and task-sharing occurred in 21 cases.	Training: AMOs were COs (3 years of medical training) who had received a further 2 years of clinical training, including a 3-month rotation in surgery and a 3-month rotation in obstetrics. Following qualification, AMOs were officially licenced to practise medicine and surgery, including performing caesarean sections. Level of supervision: AMOs in the intervention group operated independently.	Fatal outcomes, including maternal deaths and perinatal deaths and major postoperative complications. ‘Quality indicators’ included ‘lack of absolute maternal or foetal indication’, ‘urgent blood need, but no transfusion’ and ‘over 3 hours to urgently indicated operation’.	No difference between AMOs and MOs in maternal mortality and perinatal mortality rates.	Equivalence between AMOs and MOs for all major postoperative complications. No difference in ‘quality indicators’.
Mpirimbanyi *et al.*, 2017. Rwanda (sub-Saharan Africa). Low-income economy.	Retrospective, cross-sectional study. Data were collected between January 2015 and December 2015.	Multi-centre study. 3 district hospitals, run by the Rwandan Ministry of Health and by a USA-based NGO.	Surgical task-shifting. Patients underwent general surgery to treat the following emergency conditions: acute abdominal conditions (14.3%), complicated hernias (7.9%), soft tissue infections (71.6%), urological emergencies (5.3%) and thoracic emergencies (0.9%).	GPs: non-specialist physicians. GPs operated on 74/122 (60.7%) patients. General surgeon: specialist physician, who operated in Butaro District Hospital. The general surgeon operated on 48/122 (39.3%) patients.	The background training of GPs was not described. No training received as part of this study. Level of supervision: in Kirehe and Rwinkwavu District Hospitals, the GPs operated independently, as they were the only operators available. In Butaro District Hospital, GPs were supported by the general surgeon.	Clinical outcomes (patient dead, discharged or recommended for transfer). In-hospital postoperative complications (any, surgical site infection, unplanned reoperation, wound dehiscence and cardiac arrest) and length of hospital stay.	There were no differences in mortality rates between patients treated by GPs or by the general surgeon.	There were no differences in presence of postoperative complications, types of postoperative complications, clinical outcomes and length of hospital stay between the GPs and the general surgeon.
Ngcobo *et al.*, 2018. South Africa (sub-Saharan Africa). Upper-middle-income economy.	Retrospective review of patient files. Data were collected over 16 months, commencing in January 2014.	Multi-centre study. All clinics and hospitals in Tshwane District providing free VMMC. All centres were supported by the Centre for HIV/AIDS Prevention Studies (CHAPS).	Surgical task-shifting. 4849 patients underwent surgical VMMC.	Clinical associates (CAs): non-physicians. CAs performed 4300 procedures (88.7%). Doctors: non-specialist physicians. Doctors undertook training to perform VMMCs delivered through CHAPs. Doctors performed 549 (11.3%) procedures.	Background training: CAs undertook a training programme delivered by CHAPs to perform VMMC. Training covered theoretical and practical aspects of VMMC procedures. Level of supervision: CAs operated independently.	Intraoperative adverse events and postoperative adverse events, including swelling, pain, bleeding, infection and wound destruction (within 2 days of the VMMC procedure). Operative duration of VMMC procedures was recorded.		Operative duration was significantly shorter for CAs than for doctors (*t* = −7, 46 minutes; *P*< 0.001). Equivalence between ACs and doctors for intraoperative and postoperative adverse events.
Ouédraogo *et al.*, 2015. Burkina Faso (sub-Saharan Africa). Low-income economy.	Prospective, descriptive and analytical study. Data were collected between February and October 2013.	Single-centre study. 1 district hospital, serving both rural and urban catchment areas.	Surgical task-shifting. 541 women underwent caesarean sections, 46 (7.7%) women underwent laparotomies and 14 (2.3%) women underwent repair of traumatic lesions. Major surgery; emergency operations.	Surgical nurses: non-physicians. Surgical nurses performed 34.8% of major obstetric procedures. Obstetricians: specialist physicians. Obstetricians performed 65.2% of major obstetric procedures.	Background training: Surgical nurses received surgical training to manage obstetric complications, including caesarean sections, emergent laparotomy for extra-uterine pregnancy or uterine rupture and sutures of traumatic lesions. Level of supervision: the level of supervision received by surgical nurses was not specified in the study.	Maternal mortality and morbidity, neonatal mortality and operative duration for caesarean section and laparotomy.	Caesarean sections. Neonatal mortality: 2.6% (surgical nurses) vs 6.6% (obstetricians), *P* = 0.043. No difference in maternal mortality between surgical nurses and obstetricians. Laparotomies. No differences in maternal or neonatal mortality rates between surgical nurses and obstetricians.	Caesarean sections and laparotomies. Median operative duration: 31.2 minutes (surgical nurses) vs 28.1 minutes (obstetricians), *P* = 0.001. Equivalent morbidity outcomes between surgical nurses and obstetricians.
Ramdas *et al.*, 2018. The Netherlands. High-income economy.	Retrospective cross-sectional study. Data were collected between 2008 and 2014.	Multi-centre study. Primary and secondary healthcare settings.	Surgical task-shifting. 2986 basal cell carcinoma excisions. Minor surgical procedures.	231 GPs: non-specialist physicians. GPs performed 931 basal cell carcinoma excisions. 22 plastic surgeons: specialist physicians. Plastic surgeons performed 1040 basal cell carcinoma excisions. 22 dermatologists: specialist physicians. Dermatologists performed 1015 basal cell carcinoma excisions.	Training and supervision: GPs were generalist doctors, with no medical or surgical specialization. They operated independently.	Number of completely excised basal cell carcinomas and risk of incomplete basal cell carcinoma excision.Comparisons between GPs and dermatologists.		Rate of complete excisions: 70% (GP) vs 93% (dermatologist): (*P* < 0.001). Risk of incomplete excision (GP vs dermatologist): OR 6.2 (4.6–8.4), *P* < 0.0001.
Rech *et al.*, 2014. The study was conducted in Kenya, South Africa, Tanzania and Zimbabwe (sub-Saharan Africa). Lower-middle- and upper-middle-income economies. Task-shifting was only practised in Tanzania.	Prospective, cross-sectional study. Data were collected in 2011 and 2012.	Multi-centre study. In Tanzania, 14 healthcare sites in 2011 and 29 sites in 2012 were visited. These included fixed, outreach and mobile healthcare sites.	Surgical task-shifting. VMMCs. In Tanzania, 128 VMMCs were performed in 2011 and 251 in 2012.	NPCs, including nurses, COs or AMOs. In Tanzania, NPCs performed 116 VMMCs (91%) in 2011 and 239 VMMCs in 2012 (95%). MDs: non-specialist or specialist physicians. In Tanzania, physicians performed 12 VMMCs (9%) in 2011 and 12 VMMCs in 2012 (5%).	Training: the background training of NPCs was not described in detail. Level of supervision: the majority of NPCs in Tanzania operated independently. They completed all suturing in 84% of cases in 2011 and in 97% of cases in 2012.	Surgical efficiency was measured in relation to two outcome variables: ‘primary provider time with client’ and ‘total elapsed operating time’.		No association between provider type and surgical efficiency outcomes.
Tariku *et al.*, 2019. Ethiopia (sub-Saharan Africa). Low-income economy.	Retrospective cohort study. Data were collected between July 2015 and June 2016.	Multi-centre study. 1 general hospital and 1 district hospital. Both hospitals satisfied Comprehensive Emergency Obstetric and Newborn Care (EmONC) standards.	Surgical task-shifting. 474 caesarean sections. Major surgical procedures; mostly emergency surgeries (92%).	Non-physician surgeons (NPSs): non-physicians. NPSs performed 388 procedures. Physicians: non-specialist and specialist physicians. They comprised 8 GPs and 78 obstetricians. Physicians performed 85 procedures.	Background training: NPSs were BSc health officers or BSc nurses who had undertaken 4 years of training on integrated emergency surgery and obstetrics. Level of supervision: the level of supervision received by NPSs was not specified.	Immediate newborn outcomes (composite measure): death or live birth with distress.	No significant differences between NPSs and physicians.	No significant differences between NPSs and physicians.
Thevi *et al.*, 2017. Malaysia (East Asia and Pacific). Upper-middle-income economy.	Retrospective analysis of patient charts. Data were collected between 2007 and 2014.	Single-centre study. Melaka Hospital, a government-funded, specialist referral hospital in Malacca.	Surgical task-shifting. 12 992 cataract surgeries. Minor surgery.	MOs: non-specialist physicians. They performed 1498 cataract surgeries. Gazetting specialists: physicians specialized in ophthalmology who had to work under the supervision of senior specialists. They performed 759 cataract surgeries. Specialists: opthalmologists who were authorized to operate independently. They performed 10 720 cataract surgeries.	Training: MOs had completed medical school, but they had not undertaken any further specialization. Level of supervision: MOs had to be supervised by a specialist surgeon during surgery.	Intraoperative complications: including posterior capsular rupture (PCR) without vitreous loss, PCR with vitreous loss, central corneal oedema, zonular dehiscence, dropped nucleus and suprachoroidal haemorrhage. Visual outcomes: best-corrected visual acuity, measured 6 weeks postoperatively. Visual acuity was classified as good, impaired or poor vision.		Intraoperative complications: 11.7% of patients operated on by MOs experienced intraoperative complications as opposed to 5.1% of patients treated by specialist ophthalmologists (*P* < 0.001). Poor or impaired visual outcomes: 18.8% (MOs and gazetting specialists) vs 15.7% (specialists) (OR 1.25, 1.09–1.42, *P* < 0.001).
Tobome *et al.*, 2021. Benin (sub-Saharan Africa). Lower-middle-income economy.	Observational, retrospective and descriptive study. Data were collected between March 2018 and November 2019.	**S**ingle-centre study. Referral and general hospital, which lacks an intensive care unit, a computed tomography scan and a microbiological laboratory.	Surgical task-shifting. Laparotomy for acute generalized peritonitis (AGP). Major, emergency general surgery. The main causes of AGP were ileal perforation (63.5%), gastric-duodenal ulcer perforation (14.3%), appendicular (7.9%) and cryptogenetic (7.9%).	GP: non-specialist physician. The GP performed 46 laparotomies (73%). General surgeons: specialist physicians. General surgeons performed 17 laparotomies (27%).	Background training: the GP had completed medical school and had 3 years of work experience. He had acquired surgical skills by working in other African and European hospitals. Level of supervision: the GP operated independently.	Mortality, morbidity and postoperative hospital length of stay.	No difference in mortality rates between the GP and the general surgeons.	Equivalent morbidity and hospital length of stay between the GP and the general surgeons.
Tyson *et al.*, 2014. Malawi (sub-Saharan Africa) Low-income economy.	Retrospective case–control study. Data were collected between January and December 2012.	Single-centre study. A tertiary referral hospital in the central region of Malawi.	Surgical task-shifting. 1004 paediatric patients underwent 1186 paediatric surgical procedures (excluding orthopaedic surgery). Minor surgical operations (31%) and major surgical operations (57%). Elective (66%) and emergency (32%) operations. The most common operative cases included congenital surgery (23%), trauma and burns (14%), ear, nose, throat (13%), general surgery (13%) and soft tissue (12%).	COs: non-physicians. COs performed 475 (40%) paediatric surgical operations. MDs: specialist physicians, comprising surgical residents and consultant surgeons. MDs performed 566 (48%) paediatric surgical operations.	Training: COs had completed the Diploma in Clinical Medicine (3 years of didactic education and a 1-year clinical internship at a central or district hospital). Upon completion, COs were licenced to practise independently. Level of supervision: COs operated independently or with the support of a specialist.	Mortality, complications, reoperation rates and hospital length of stay.	No difference in mortality rates between COs and MDs.	No difference in complication rates between COs and MDs, even when stratified by case complexity (minor or major surgery). Reoperation rate: 17% (COs) vs 7.1% (MDs), *P* < 0.001. Hospital length of stay: 24 days (COs) vs 10 days (MDs), *P* < 0.001. The authors noted that reoperation rates and hospital length of stay did not differ significantly after controlling for burn cases.
Wamalwa *et al.*, 2015. Kenya (sub-Saharan Africa). Lower-middle-income economy.	Prospective (cohort) study. Data were collected over an 18-month period.	Single-centre study. General surgical unit at Garissa Provincial General Hospital.	Surgical task-shifting. Inguinal hernia repair, utilizing the Shouldice or the Lichtenstein surgical techniques. Major general surgery; elective procedures.	MO: non-specialist physician. The MO operated on 25 patients. General surgeon: specialist physician. The general surgeon operated on 20 patients.	Background training: the MO was a generalist doctor with no postgraduate specialization. Study training: the MO was trained to perform hernia repair using the Shouldice and the Litchenstein techniques in a standard manner. Level of supervision: the MO operated independently.	Operative time (minutes), number of technically difficult operations and postoperative complications (at 24 hours and 2 weeks post-surgery).		No difference in outcomes between the MO and the general surgeon.
Wilhelm *et al.*, 2011. Malawi (sub-Saharan Africa). Low-income economy.	Retrospective review of theatre books. Data were collected between January 2003 and December 2007.	Single-centre study. Zomba Central Hospital, Department of Surgery and Orthopaedics. Teaching hospital that serves as the district hospital for the Zomba District and as the referral hospital for the south-eastern zone. It serves a catchment population of ∼2.6 million people.	Surgical task-shifting. Transvesical prostatectomy for benign prostate hyperplasia, ventriculoperitoneal shunting for hydrocephalus and hernia repair with bowel resection and anastomosis for strangulated hernia were selected for outcome analysis.	COs: non-physicians. In total, COs performed 61 VP shuntings, 113 trasvesical prostatectomies and 21 hernia repairs. General surgeons: specialist physicians. ‘Surgeon present’ operations included 51 VP shuntings, 101 transvesical prostatectomies and 32 hernia repairs.	Background training of COs: 3-year pre-service training followed by a 1-year internship. The internship included a 3-month rotation in general surgery and obstetrics and gynaecology. Surgical skills were further developed through on-the-job instruction by supervisors. Level of supervision: COs in the study intervention group operated independently.	VP-shunting: mortality (perioperative), wound infection, postoperative hospital length of stay, shunt revision and shunt explant. Transvesical prostatectomy: mortality (perioperative), wound infection, bladder leakage, postoperative hospital length of stay, postoperative blood transfusion and reoperation. Hernia repair: mortality (perioperative), wound infection, postoperative hospital length of stay, reoperation and anastomotic leakage.	VP-shunting. No difference in mortality rates between COs and general surgeons. Transvesical prostatectomy. No difference in mortality rates between COs and general surgeons. Hernia repair. No difference in mortality rates between COs and general surgeons.	VP-shunting. Hospital length of stay: 10 days (4–15) (COs) vs 8 days (3–35) (surgeons), *P* = 0.03. No significant differences for other morbidity measures. Transvesical prostatectomy. Hospital length of stay: 16 days (9–74) (COs) vs 15 days (7–38) (surgeons), *P* = 0.05. Postoperative blood transfusion: 19.5% (COs) vs 9.9% (surgeons), *P* = 0.06. No differences between COs and surgeons in other morbidity measures. Hernia repair. No significant differences between COs and surgeons for morbidity outcomes.
Wilhelm *et al.*, 2017. Malawi (sub-Saharan Africa). Low-income economy.	Retrospective comparative study. Data were collected between January 2006 and December 2010.	Single-centre study. Zomba Central Hospital, Department of Surgery and Orthopaedics. Teaching hospital that serves as the district hospital for the Zomba District and as the referral hospital for the south-eastern zone. It serves a catchment population of ∼2.6 million people.	Surgical task-shifting. Major amputations (108 procedures) and open reductions and plating of fractured long bones (155 procedures). Major orthopaedic surgeries.	Orthopaedic clinical officers (OCOs): non-physicians. OCOs performed 71.3% of major amputations and 38.7% of internal fixations of long bones with plates. International surgeons: specialist physicians. All surgeons were general surgeons; one had also trained in orthopaedic surgery. They performed 28.7% of major amputations and 61.3% of internal fixations of long bones with plates.	Training: OCOs had ∼4 years of work experience as medical assistants and had completed the Diploma in Clinical Orthopaedics, an 18-month training course from the Malawi College of Health Sciences. Before being licenced to work independently, OCOs had operated under close supervision for several years. Level of supervision: OCOs in the intervention group operated independently.	Major amputations. Perioperative mortality, postoperative blood transfusion, postoperative infection, reoperation rates and postoperative hospital length of stay. Open reduction and internal fixations with plates. Postoperative infection, reoperation rates, other complications and postoperative hospital length of stay.	Major amputations. There were no differences in mortality rates between OCOs and surgeons. Open reduction and internal fixations with plates.	Major amputations. Equivalent postoperative blood transfusion, postoperative infection, reoperation rates and hospital length of stay. Open reduction and internal fixations with plates. Equivalent postoperative infection, reoperation rates, other complications and hospital length of stay.
Van Duinen *et al.*, 2019. Sierra Leone (sub-Saharan Africa). Low-income economy.	Prospective, observational, multi-centre, non-inferiority study.	Multi-centre study. 9 public and private hospitals across Sierra Leone: 4 were public district hospitals, 1 was a public regional hospital, 1 was a public tertiary hospital and 3 were private non-profit hospitals. Data were collected between October 2016 and May 2017.	Surgical task-shifting. 1282 caesarean sections, including laparotomy for uterine rupture. Major operations. Prevalent indications: obstructed and prolonged labour (ACs 55.5%, MDs 54.6%), previous caesarean section (ACs 15.1%; MDs 11.7%), antepartum haemorrhage (ACs 10.8%; MDs 12.3%) Emergency caesarean sections: 81.3% of caesarean sections performed by ACs; 88.9% of caesarean sections performed by MDs.	ACs: non-physicians. 11/12 ACs were trained in Sierra Leone. ACs performed 444 caesarean sections. Of these, 443 caesarean sections were analysed. MDs: specialist and non-specialist physicians. 25/50 MDs were trained in Sierra Leone.	Background and study training: CHOs with 2 years of work experience could undertake a task-sharing/shifting training programme to qualify as ACs. A 2-year training programme to manage basic emergency surgical and obstetrical conditions. 4 ACs (33%) had <1 year of previous work experience; 7 (58%) ACs had 1–5 years of previous work experience. Solely 1 AC (8%) had over 5 years of previous work experience. Level of supervision: ACs were the primary surgical providers for ‘intervention group’ patients.	Primary outcomes: perioperative maternal mortality (intraoperative or within 30 days of the operation). Secondary outcomes: perinatal outcomes and maternal morbidity parameters; operative time and duration of hospital length of stay. Perinatal outcomes included macerated and fresh stillbirths and neonatal deaths and perinatal deaths (the sum of fresh stillbirths and early neonatal deaths). Maternal morbidity parameters included blood loss exceeding 600 ml, reoperation, readmission, wound infection and persistent postoperative abdominal pain.	Maternal mortality: 0.2% (ACs) vs 1.8% (MDs), adjusted OR 0.11, 0.01–0.63. Neonatal stillbirth: 12.7% (ACs) vs 16.6% (MDs), adjusted OR 0.74 95% confidence interval 0.56–0.98. There were no other significant differences in (early and late) neonatal deaths and perinatal deaths.	Readmission rates: 3.7% (ACs) vs 1.7% (MDs), adjusted OR 2.17, 1.08–4.42). Operative time: 31.9 minutes (ACs) vs 38.9 minutes (MDs), *P* < 0.001. There were no significant differences in blood loss exceeding 600 ml, reoperation, wound infection, persistent postoperative abdominal pain or duration of hospital stay.
Young and Bowling, 2012. Michigan (USA). High-income economy.	Retrospective, non-inferiority trial with a 5% point difference. Data were collected between June 2005 and March 2010.	Single-centre study. Urban, Level I trauma centre.	Surgical task-shifting. Placement of intracranial pressure (ICP) monitors as a result of traumatic brain injury. Emergency, major surgical procedure.	MLPs: non-physicians. MLPs performed 70 ICP monitor placements. Neurosurgeons: specialist physicians. They performed 22 ICP monitor placements.	Training and level of supervision: MLPs began by first assisting in the operating room and managing patients in the general surgery ward, before treating patients with trauma in the emergency department and in the critical care unit. After 4/5 years of experience, above-average MLPs were eligible for neurosurgical training. MLPs were directly supervised by a senior MLP with neurosurgical experience and by attending neurosurgeons and they had to correctly place five ICP monitors under supervision before operating independently. The decision to place ICP monitors remained the responsibility of the neurosurgeons.	Mortality and expected ratio of survivors, major complications (infection, haemorrhage and leak) and minor complications (malfunction and malposition). Time to monitor placement, duration of monitoring (days) and good Glasgow Outcome Score (score 4 or 5).	No difference in mortality rates between MLPs and neurosurgeons.	Major complications: 1.4% (MLPs) vs 0% (neurosurgeons), *P* = 0.0128 (significantly <5%). Minor complications: 5.7% vs 0%, *P* = 0.80. Duration of monitor placement: 5 days vs 7.5 days, *P* = 0.018. No difference in good Glasgow Outcome Score between provider groups.

**Table 4.** Characteristics of included studies, anaesthetic task-shifting

Study name and country	Study design	Healthcare facility characteristics	Procedure characteristics	Lead anaesthetic providers and number of procedures	Training and level of supervision of non-specialists	Outcomes assessed	Mortality	Other clinical outcomes
Cometto *et al.*, 2012. South Sudan (sub-Saharan Africa). Low-income economy.	Retrospective review. Data were collected between 2005 and 2010.	Multi-centre study. The programme was implemented in Primary Healthcare Centres, supported by the Italian NGO Comitato Collaborazione Medica.	Anaesthetic task-shifting. 1543 patients received anaesthesia during the surgical missions of Comitato Collaborazione Medica. Most operations (60%) were performed under spinal anaesthesia. A minority of cases required ketamine anaesthesia with intravenous supplementation of analgesic drugs. Many cases of minor surgery were performed under local infiltration. Few cases (e.g. goitres and acute abdomen) were operated under general anaesthesia with endotracheal intubation and ventilation with Ambu balloon.	Anaesthesia technicians: non-physicians. They administered anaesthesia to 511 surgical patients. Visiting expatriate consultant surgeons: specialist physicians. They administered anaesthesia to 1032 surgical patients.	Anaesthesia technicians had a level of background training between nurses and physicians. They were trained through the training programme of Comitato Collaborazione Medica and through the War Wounded Referral Hospital (Kenya) managed by the International Committee of the Red Cross. They developed competencies in perioperative surgical care.	Mortality.	No differences between anaesthesia technicians and expatriate surgeons.	
Dulisse and Cromwell, 2010. USA. High-income economy.	Retrospective review. Data were collected between 1999 and 2005 through the ‘Medicare Parts A and B claims’ limited datasets across ‘non-opt-out’ states and 14 ‘opt-out’ states. Opt-out states were those that allowed the reimbursement of certified registered nurse anaesthetists (CRNAs) operating independently through Medicare and Medicaid Services.	Multi-centre study.	Anaesthetic task-shifting. Anaesthesia was administered as part of surgical procedures.	CRNAs practicing independently (CRNA solo): non-physicians. CRNAs independently provided anaesthesia in 21% of surgeries in opt-out states and in 9.7% of surgeries in non-opt-out states between 1999 and 2005. Medical Doctor Anaesthesiologists (MDA) practicing independently : specialist physicians. They independently provided anaesthesia in 42% of surgeries in opt-out states and in 44.5% of surgeries in non-opt-out states between 1999 and 2005. Team (MDAs and CRNAs): teams provided anaesthesia in 37% of surgeries in opt-out states and 45.8% of surgeries in non-opt-out states between 1999 and 2005.	The training of CRNAs was not described in detail.	In-patient mortality and anaesthesia complications. Outcomes were calculated taking the ‘MDA solo’ group in non-opt-out states as the reference category. To evaluate task-shifting, authors focused on the comparisons between the ‘MDA solo’ group in non-opt-out states group and ‘CRNA solo’ groups (in non-opt-out and opt-out states).	CRNA solo’ in non-opt-out states: OR 0.899 (*P* = 0.05). ‘CRNA solo’ in opt-out states, before opting out: OR 0.651 (*P* = 0.05). ‘CRNA solo’ in opt-out States, after opting out: OR 0.689 (*P* = 0.05).	CRNA solo’ in non-opt-out states: OR 0.992 (not significant). ‘CRNA solo’ in opt-out states, before opting out: OR 0.798 (*P* ≤ 0.05). ‘CRNA solo’ in opt-out states, after opting out: OR 0.813 (*P* ≤ 0.05).
Kudsk-Iversen *et al.*, 2020. 23 countries across the Africa Region, the Americas Region, the Eastern Mediterranean Region and the South-East Asia Region.	Retrospective observational study. Data were collected between January 2008 and December 2017.	Multi-centre study. Data were collected for 173 084 cases across 23 countries and 52 different locations. 28 surgical projects were set in armed conflict settings and 32 projects were in healthcare gap (HG) settings.	Anaesthetic task-shifting. Anaesthesia was administered as part of surgical projects delivered by Médecins Sans Frontières (MSF). Most common anaesthesia: spinal injection alone and general anaesthesia without intubation (mostly ketamine based).	MSF projects were classified based on the most senior anaesthetic provider. Uncertified anaesthesia providers: local non-physicians. They led 15% of cases in armed conflict settings and HG settings. Nurse anaesthetists: nurses or other non-physician clinical cadres who were predominantly from low-income settings. They led 19% of cases in armed conflict settings and HG settings. Anaesthesiologists: specialist physicians with qualifications in anaesthesia, either local or expatriates (from both low-income and high-income settings). They led 66% of cases in armed conflict settings and HG settings.	Uncertified anaesthesia providers had different levels of experience in anaesthesia provision, but they lacked formal qualifications. They received on-the-job training. Nurse anaesthetists had received formal training and qualification in anaesthesia in their country of origin.	Mortality (intraoperative).	Comparable mortality rates between provider groups. Armed conflict settings: 0.3% (uncertified providers), 0.2% (nurse anaesthetists) and 0.3% (anaesthesiologists). HG settings: 0.2% (uncertified providers), 0.1% (nurse anaesthetists) and 0.3% (anaesthesiologists).	
Needleman and Minnick, 2009. USA (California, Florida, Kentucky, New York, Texas, Washington and Wisconsin). High-income economy.	Retrospective review. Data were collected in 1999–2001 (California, Florida, New York, Washington and Wisconsin) and 2000–2001 (Kentucky and Texas).	Multi-centre study across 369 hospitals. 27% of hospitals were located in California, 19% in Wisconsin, 14% in New York, 13% in Texas, 13% in Washington, 9% in Florida and 5% in Kentucky. 67% were metropolitan hospitals, 83% were non-teaching hospitals and 69% were non-profit hospitals.	Anaesthetic task-shifting. Anaesthesia was delivered for 1 141 641 obstetrical surgical procedures (caesarean section deliveries).	CRNAs only: non-physicians. Solely CRNAs administered anaesthesia in the study hospital. 23% of hospitals implemented a ‘CRNA-only’ model. Anaesthesiologists only: specialist physicians. Solely anaesthesiologists administered anaesthesia in the study hospitals. 39% of hospitals implemented an ‘anaesthesiologist-only’ model. Other models of anaesthesia provision included ‘ANES-CRNA I’, ‘ANES-CRNA II’ and ‘mixed’. To evaluate task-shifting, we focused on comparisons between the ‘anaesthesiologist-only’ and ‘CRNA-only’ groups.	The training of CRNAs was not described in detail.	Mortality (in-hospital), anaesthesia complications, anaesthesia or other complications (composite outcome) and obstetrical trauma (Agency for Healthcare Research and Quality Patient Safety indicators related to obstetrical trauma). Outcomes were calculated taking the ‘anaesthesiologist-only’ group as the reference category.	No significant differences between ‘CRNAs only’ and ‘anaesthesiologists only’ for mortality.	Anaesthesia or other complications, ‘CRNAs only’ vs ‘anaesthesiologists only’: OR 0.723 (0.542–0.965, *P* = 0.028). No significant differences between ‘CRNA-only’ and ‘anaesthesiologist-only’ groups for anaesthesia complications and obstetrical trauma.
van der Merwe *et al.*, 2021. 25 countries across Africa.	Secondary analysis of the African Surgical Outcomes Study, a prospective observational cohort study. Data were collected over 7 days, between February and May 2016.	Multi-centre study. Patients treated by non-physicians: 25% in primary healthcare facilities; 27% in secondary healthcare facilities and 49% in tertiary healthcare facilities. 83% of hospitals were government funded and 17% were privately funded or mixed. Patients treated by physicians: 22% in primary healthcare facilities, 29% in secondary healthcare facilities and 50% in tertiary healthcare facilities. 85% of hospitals were government-funded and 15% were privately funded or mixed.	Anaesthetic task-shifting. Procedural sedation administered for adult elective and emergency in-patient surgical operations. General and regional anaesthesia were excluded. Severity, by provider type: non-physicians: 50% minor, 44% intermediate and 6% major. Physicians: 53% minor, 31% intermediate and 17% major. Urgency: non-physicians: 48% emergency. Physicians: 40% emergency.	Non-physicians administered anaesthesia to 98 patients. Physicians: specialists and non-specialists. They administered anaesthesia to 235 patients.	Training and level of supervision of non-physicians were not specified.	Severe complications and death (composite outcome).	12.8% (non-physicians) vs 1.6% (physicians), average treatment effect 8.3 (2.7–25.6), *P* < 0.001.	12.8% (non-physicians) vs 1.6% (physicians), average treatment effect 8.3 (2.7–25.6, *P* < 0.001).

All studies were published between 2008 and 2021, with 24 papers (60%) published since 2015. Based on the World Bank income group classification, 17 studies were in low-income economies, 8 studies in lower-middle-income settings, 2 in upper-middle-income settings and 11 in HICs. van der Merwe *et al.* (2021) analysed the data across 25 African countries, and Kudsk-Iversen *et al.* (2020) included data collected across five WHO regions, ranging between low-income and upper-middle-income settings. Most studies came from sub-Saharan Africa (27 studies, 67.5%), followed by the USA (10 studies, 25%).

Thirty studies (75%) reported on perioperative mortality and 34 studies (85%) analysed morbidity measures, including perioperative complications, hospital length of stay and duration of the operative procedure. Fourteen articles (35%) adopted prospective study designs, including one RCT (Ashley *et al.*, 2021), whereas 26 studies (65%) were retrospective. Twenty-two publications (55%) were multi-centre studies.

### Surgical task shifting in LMICs

#### Obstetric surgery

In total, eight studies in sub-Saharan Africa assessed the impact of task-shifting for obstetric surgery: all papers compared outcomes of caesarean sections between non-physicians and physicians. In a prospective study by Gajewski *et al.* 2019b, wound infection rates for caesarean deliveries performed across eight district hospitals in Zambia were equivalent between medical licentiates and medical doctors. Gessessew *et al.* (2011) performed a retrospective study comparing caesarean delivery outcomes of 11 NPCs and 4 obstetricians in 13 Comprehensive Emergency Obstetric Care (CEmOC) facilities in Tigray Region, Ethiopia. NPCs performed 63.3% of all obstetric surgical interventions and 55.9% of emergency caesarean sections; they were associated with equivalent rates of maternal deaths, foetal deaths and hospital length of stay as specialist physicians. In Tanzania, the safety of caesarean sections performed by assistant medical officers and medical doctors was not significantly different, based on maternal mortality, maternal complications and early neonatal mortality measures. However, Matinhure *et al.* (2018) did highlight that physicians were significantly more likely to perform emergency deliveries [odds ratio (OR) 2.52, 1.80–3.53].

Ouédraogo *et al.* (2015) noted that surgical nurses were not inferior to obstetricians when comparing maternal mortality, maternal morbidity and neonatal mortality (2.6% vs 6.6%, *P* = 0.043). Similarly, Tariku *et al.* (2019) reported no significant differences in ‘perinatal mortality and surgical complications’ between non-physicians and physicians performing caesarean sections in two CEmOC hospitals in Ethiopia. In a study by (Van Duinen *et al.* 2019), despite higher readmission rates (3.7% vs 1.7%; OR 2.17, 1.08–4.42), caesarean sections performed by associate clinicians were shown to be safe, comparing favourably to physicians for maternal mortality (0.2% vs 1.8%; OR 0.11, 0.01–0.63) and stillbirths (12.7% vs 16.4%; OR: 0.74, 0.56–0.98). Blood loss, wound infection, postoperative pain and reoperation were not related to provider cadre and associate clinicians reported shorter operative times (31.9 vs 38.9 minutes, *P* < 0.001). McCord *et al.* (2009) reported no significant differences in mother or child fatal outcomes following emergency caesarean sections performed by assistant medical officers and medical officers in 14 hospitals across two regions of Tanzania. Additionally, there were no significant differences in major complications and quality indicators between the two patient groups. Instead, significant differences in patient outcomes were measured between government hospitals and mission hospitals.

In a retrospective, cross-sectional study in Burkina Faso, Hounton *et al.* (2009) compared the quality of caesarean sections performed by clinical officers, GPs and obstetricians. Although no significant differences were noted in maternal case fatality rates and maternal morbidity measures, newborn case fatality rates were higher for clinical officers as opposed to non-specialist and specialist physicians (198/1000 vs 125/1000 vs 99/1000). Furthermore, operative times and postoperative hospital length of stay were, respectively, 20% and 30% shorter for obstetricians.

#### Hernia repairs

In total, nine studies in sub-Saharan African countries addressed task-shifting for general surgery procedures. Most studies focused on elective or emergent hernia repairs: four studies compared the performance of non-physicians with medical doctors, whereas two studies compared non-specialist physicians and qualified surgeons. Ashley *et al.* (2021), the only RCT identified in this systematic review, compared the outcomes of hernia repair with mesh when performed by six associate clinicians and five medical doctors in Sierra Leone. At 1-year follow-up, there were no differences in mortality rates; solely one (0.9%) hernia recurrence was reported in the intervention group against seven (6.9%) in the control arm, demonstrating the non-inferiority of treatment by associate clinicians (*P* < 0.001). Similarly, there was an equivalent distribution of secondary outcomes at 2 weeks (postoperative complications) and 1 year post-surgery (fewer groin symptoms, pain, patient satisfaction and self-assessed health status). In a retrospective study across seven healthcare facilities in the Pwani Region of Tanzania, Beard *et al.* (2014) noted that clinical officers and assistant medical officers were the primary surgical providers in 87.1% of district hospitals and 67.2% of mission hospitals: overall, they performed 81.8% of elective hernia repairs and 81.2% of emergent hernia repairs. When comparing mortality and morbidity outcomes for NPCs and physicians, no significant differences between the two provider cadres were detected. Wilhelm *et al.* (2011) (retrospective) and Gajewski *et al.* (2019a) (prospective) studied the outcomes of inguinal hernia repairs undertaken by clinical officers and physicians in Malawi. Both papers concluded that there were no statistically significant differences in wound infection outcomes. Additionally, Wilhelm *et al.* (2011) reported comparable outcomes for mortality, anastomotic leakage, and reoperation rates and duration of hospital stay.

According to Beard *et al.* (2019), in a regional hospital in Ghana, three medical officers were deemed to be as safe as two general surgeons performing elective inguinal hernia repair with mesh. The former providers were non-inferior based on mortality and hernia recurrence after 1 year (0.9% vs 2.8%, absolute difference −1.9, *P* < 0.01) as well as secondary outcomes at 2 weeks and 1 year post-surgery. Similarly, in a prospective study in Kenya, Wamalwa *et al.* (2015) found that medical doctors were equivalent to surgeons when undertaking hernia repairs using the Lichtenstein and the Shouldice techniques. Outcomes studied comprised postoperative complications at 24 hours and 2 weeks post-surgery, operative time and number of technically difficult operations performed.

#### Other general surgery

Beyond hernia repairs, Beard *et al.* (2014) found no significant differences in mortality and morbidity outcomes for other general surgery operations performed by NPCs and physicians. The most common procedures included prostatectomy, exploratory laparotomy and hydrocelectomy. In Gajewski *et al.* 2019b, wound infection rates for index general surgery procedures were equivalent for medical licentiates and medical doctors operating in Zambia. In addition to hernia repairs, Wilhelm *et al.* (2011) studied outcomes of ventriculo-peritoneal shunting (VP-shunting) for hydrocephalus and transvesical prostatectomy for benign prostate hyperplasia, which were led by clinical officers or by general surgeons in a referral and teaching hospital in Malawi. For VP shunting, rates of mortality and wound infection, early shunt revision and shunt explants were not statistically different between the two provider groups; however, average postoperative hospital stay was significantly shorter in the surgeon group (10 vs 8 days, *P* = 0.03). Analogously, postoperative hospital stay was longer for the intervention group (16 vs 15 days, *P* = 0.05) in the case of prostatectomy; no other outcome parameter differed between the two groups of operators (mortality, wound infection, bladder leakage or reoperation).

Tobome *et al.* (2021) conducted a retrospective study in a peripheral, referral hospital in Western Benin where a GP and two general surgeons treated patients with acute generalized peritonitis. The GP performed 73% of operations, with mortality rates and postoperative complication rates analogous to the specialist physicians. In a retrospective study, Mpirimbanyi *et al.* (2017) assessed the clinical outcomes of all patients presenting to three rural district hospitals in Rwanda with emergency general surgical conditions. No significant differences between patients treated by a GP as opposed to a general surgeon were highlighted when assessing mortality, postoperative complications and hospital length of stay.

#### Surgical male circumcision

Four studies assessed outcomes of surgical male circumcisions undertaken by NPCs or physicians in sub-Saharan African settings (Buwembo *et al.*, 2011; Rech *et al.*, 2014; Ngcobo *et al.*, 2018; Matumaini *et al.*, 2021). All studies demonstrated the comparability of the lower-level cadres with the standard practice: there were no significant differences in the rates of perioperative adverse events (including bleeding, infection, wound dehiscence, swelling and pain) and in operative timings between provider types. Interestingly, Buwembo *et al.* (2011) highlighted that in a multivariate regression analysis surgical outcomes were significantly associated with provider experience, independently of professional title: increased surgical experience of the provider reduced operative duration by 1.5 minutes (*P* < 0.001).

#### Other surgical procedures in LMICs

Attebery *et al.* (2010) reported on an assistant medical officer who was trained to provide essential neurosurgical services in a rural referral hospital in Tanzania. When comparing his performance with that of an American neurosurgeon, no differences in mortality rates were detected. Through a prospective observational study, Bolkan *et al.* (2017) sought to analyse the performance of trainees and graduates [Surgical Assistant Community Health Officers (SACHOs)] of a surgical training programme, promoted by the non-profit organization CapaCare and by the Ministry of Health in Sierra Leone. Adjusted mortality rates between ‘indirectly supervised’ and ‘observed’ surgical and obstetric procedures performed by trainees (0.8% vs 2.6%, OR 0.47, 0.32–0.71, *P* < 0.001) and by SACHOs (0.8% vs 9.6%, OR 0.16, 0.07–0.41; *P* < 0.001) proved the safety of task-shifting through this programme.

Chu *et al.* (2011) described the outcomes of all surgical interventions taking place in a private hospital in Guri-El, central Somalia. Procedures were performed by specialist surgeons from Médecins Sans Frontières until January 2008 and by a local general doctor with surgical skills in conjunction with a surgical nurse between January 2008 and December 2009. Perioperative mortality was higher between 2006 and 2009 than in 2008–2009 (1.7% vs 0.2%, *P* < 0.001). In a study by Cometto *et al.* (2012), no statistically significant differences were found in mortality rates following 1543 surgical operations performed by surgical technicians or by visiting consultant surgeons in Primary Health Care Centres in Southern Sudan.

Tyson *et al.* (2014) conducted a case–control study of paediatric surgery operations performed in a tertiary referral hospital in central Malawi by clinical officers as opposed to consultant surgeons and surgical residents. The NPCs led ∼40% of all cases, and they most commonly performed burn surgery, neurosurgery and ear, nose, throat procedures. Overall, mortality and complication rates stratified by case complexity were similar between clinical officers and specialists. The authors initially noted longer duration of hospital length of stay (24 vs 10 days, *P* < 0.001) and higher reoperation rates (17% vs 7.1%, *P* < 0.001) in patients operated on by clinical officers although these differences ceased after controlling for burn victims.

Wilhelm *et al.* (2017) compared orthopaedic clinical officers and surgeons, performing major amputations and open reduction and internal fixations in Zomba Central Hospital, Malawi. The baseline characteristics of patients were comparable between provider cadres and no significant differences in mortality or postoperative complications emerged, including infection rates, postoperative blood transfusion, reoperation and postoperative hospital length of stay.

In an 8-year retrospective study in Melaka Hospital (Malaysia), Thevi *et al.* (2017) studied the relationship between the grade of operators and the occurrence of complications during cataract surgery: 11.7% of patients operated on by medical officers (non-specialists) experienced an intraoperative adverse event as opposed to 5.1% of patients treated by specialist ophthalmologists (*P* < 0.001).

### Surgical task-shifting in HICs

#### Tube thoracostomy

Two studies focused on tube thoracostomies in the USA. Bevis *et al.* (2008) sought to compare the quality of tube thoracostomies done by advanced practice providers (advanced registered nurse practitioners and physician assistants) and by trauma surgeons. Overall, no differences were identified in insertion complications, outcome complications requiring additional intervention or hospital length of stay. The only significant difference between practitioner types was ‘complication when the thoracostomy tube extended caudad from the insertion site’ (2.6% vs 21%, *P* = 0.02). No deaths occurred as a direct result of the tube thoracostomies. In a study by Harrell *et al.* (2020), thoracostomy tubes were placed by aeromedical non-physicians in a pre-hospital setting or by physicians in a hospital setting. There were no differences in mortality or complication rates. Although patients treated by non-physicians were more likely to be disposed to the intensive care unit (ICU) (67.3% vs 34.0%, *P* = 0.006) and experience significantly longer ICU length of stay (10.35 vs 6.70 days, *P* = 0.034), there were no differences in overall hospital length of stay, ventilator days and hospital disposition. Overall, in both papers, advanced practice providers were proven to be safe providers of tube thoracostomies.

#### Neurosurgery

Three publications discussed neurosurgical procedures. Enriquez-Marulanda *et al.* (2018) and Ellens *et al.* (2019) compared placements of external ventricular drains (EVDs) by trained advanced practice providers and physicians. Across both articles, the authors found no significant differences in placement accuracy, postoperative Glasgow Coma Score, and intraoperative and postoperative complications, including haemorrhage, infection and catheter leaks. In Ellens *et al.* (2019), 18 patients treated by non-physicians required multiple passes of the catheter for correct EVD placement, without further complications. Enriquez-Marulanda *et al.* (2018) observed that non-physicians had a higher risk of non-functional EVD (21.8% vs 11.9%, *P* = 0.04). Young and Bowling (2012) studied patient outcomes following intracranial pressure monitor positioning by mid-level providers or by neurosurgeons. The difference in major complication rates was significantly <5% (1.4% vs 0%, *P* = 0.0128); instead, there was a clinically significant difference in minor complication rates (5.7% vs 0%, *P* = 0.80).

#### Surgical male circumcision

Two studies from the USA focused on surgical circumcisions by non-physicians in the paediatric population. Gerber *et al.* (2019) did not identify any significant difference between advanced practice providers and urologists performing the procedure in terms of overall complications, revision of circumcision or need for penile surgery following circumcision and 30-day return to the emergency department . In Giramonti and Kogan (2018), operative times were comparable between advanced practice providers and urologists, with 63% of ‘non-physician’ cases and 46% of ‘physician’ cases taking 21–30 minutes to complete.

#### Obstetric surgery

Homan *et al.* (2013) sought to compare the outcomes of caesarean deliveries performed by family physicians or by obstetricians in two rural community hospitals in New England, USA. Although the family medicine hospital recorded longer average procedure times (55.2 vs. 42.5 minutes, *P* < 0.01) and maternal hospital length of stay (3.0 vs 2.6 days, *P* < 0.01), there was no evidence of increased risk for mothers and newborns. During the study period, no maternal deaths occurred in either healthcare facility and no statistically significant differences were found for intraoperative and infectious complications and newborn outcomes. Overall, mothers operated on by family physicians experienced fewer maternal postoperative complications (0.03 vs 0.12, *P* = 0.03).

#### Basal cell carcinoma excision

Finally, Ramdas *et al.* (2018) analysed pathology records of excisions of basal cell carcinomas performed by GPs as opposed to dermatologists or plastic surgeons in the Netherlands between 2008 and 2014. The rates of complete excisions were significantly lower for GPs as opposed to the dermatologists (70% vs 93%, *P* < 0.001), with the risk of incomplete excision being six times higher for procedures completed by a GP (OR 6.2, 4.6–8.4, *P* < 0.0001).

### Anaesthetic task-shifting globally

Five papers implemented a comparative approach to study patients’ clinical outcomes following the administration of anaesthesia or sedation for surgical procedures. Three studies were set in LMICs and two in the USA. All studies compared the performance of non-physicians with that of qualified anaesthesiologists.

Cometto *et al.* (2012) reported on anaesthesia provided by anaesthesia technicians (non-physicians) and by consultant anaesthetists during surgical missions of the NGO Comitato Collaborazione Medica in Southern Sudan. Operations were mostly undertaken under spinal anaesthesia (60%), whereas ketamine and general anaesthesia with endotracheal intubation were used in a minority of cases. The total mortality rate was 0.58%, and no significant differences between provider groups were identified. Kudsk-Iversen *et al.* (2020) examined the safety of anaesthesia care provided by Médecins Sans Frontières in crisis settings across 23 countries between January 2008 and December 2017. In active armed conflict situations, mortality rates were 0.3%, 0.2% and 0.3% for uncertified providers, nurse anaesthetists and anaesthesiologists, respectively; in poor-resourced contexts experiencing healthcare gaps, the mortality rates were 0.2% for uncertified providers, 0.1% for nurse anaesthetists and 0.3% for physician anaesthesiologists (van der Merwe *et al.*, 2021) sought to describe the outcomes of 336 procedural sedations for adult elective and emergency surgery, administered by non-physician or physician anaesthesia providers across 25 African countries. Through an inverse probability of treatment-weighted model, the authors estimated the average treatment effect of exposure to a non-physician provider: they detected an 8-fold increase in the odds of ‘severe complications and death’ in patients treated by non-physicians (12.8% vs 1.6%; adjusted OR 8.3, 2.7–25.6).

Two publications were set in the USA. Needleman and Minnick (2009) studied four outcome measures to establish the safety of nurse anaesthetists providing anaesthesia for obstetric surgery. Taking the ‘anaesthesiologist-only’ group as the reference category, nurse anaesthetists operating independently were not inferior based on mortality, anaesthesia complications, other complications and obstetrical trauma indicators. Analogous results were shown for nurse anaesthetists operating under the supervision of physicians. Dulisse and Cromwell (2010) assessed the clinical outcomes of patients who underwent anaesthesia between 1999 and 2005 in states where nurse anaesthetists could operate without supervision (opt-out states). The reference group was the ‘anaesthesiologist solo group’ in non-opt-out states. Nurse anaesthetists operating independently in opt-out states were not inferior to the control group for mortality (OR 0.689, *P* = 0.05) and complications (OR 0.813, *P* = 0.05).

## Discussion

Our review identified 40 studies assessing patient outcomes because of anaesthetic and surgical task-shifting published between 2008 and 2021. All but four of the studies focused on surgical task-shifting, and the majority were conducted in sub-Saharan Africa. Only one study was an RCT, highlighting the limited high-quality evidence on the clinical implications of task-shifting.

In LMICs, the body of evidence mostly pertains to caesarean sections, general surgery, including hernia repairs, and surgical male circumcisions. Overall, evidence indicates that these common and low-complexity procedures have been performed safely by trained non-physicians and non-specialist physicians, with comparable outcomes between intervention and control groups. This aligns with existing literature that compared caesarean sections, hernia repairs and male circumcisions between non-physician and physician operators, without reporting significant differences in key outcomes (Wilson *et al.*, 2011; Ford *et al.*, 2012; Schroeder *et al.*, 2020; Zakhari *et al.*, 2022). However, we identified limited literature addressing surgical task-shifting in HICs and a lack of information on anaesthetic task-shifting with findings being generally of poor quality, rendering it precocious to draw firm conclusions. In LMICs, the current evidence on anaesthetic task-shifting appears to be conflicting, as previously highlighted by Lewis *et al.* (2014) and Sobhy *et al.* (2016). Even though two studies demonstrated similar outcomes between provider groups (Cometto *et al.*, 2012; Kudsk-Iversen *et al.*, 2020), results from a large prospective cohort study conducted across 25 African countries suggested that performance of non-anaesthesiologists was inferior to that of anaesthesiologists (van der Merwe *et al.*, 2021).

### Strengths and limitations

To the best of our knowledge, this is the most comprehensive literature review with a broad scope to synthesize the evidence of intraoperative anaesthetic and surgical task-shifting across all World Bank income groups. We employed a broad eligibility criterion with a comprehensive search strategy across four databases to capture all the existing literature examining the clinical impact of task-shifting to non-physicians or non-specialist physicians.

Our search strategy excluded any articles published prior to 2008, possibly omitting some research studies on the clinical implications of task-shifting. However, while task-shifting may have been studied in certain settings before, we focused on a period with sustained policy focus following the WHO’s first conference on task-shifting (WHO, PEPFAR, UNAIDS, 2008). A methodological limitation of this study relates to its exclusion of broader perioperative aspects of surgical care provision, beyond the technical performance of anaesthesia and surgery.

Due to the overall poor quality of the included studies, and their heterogeneous nature, we were precluded from performing a meta-analysis of the extracted data. Nonetheless, our narrative synthesis offers broad insights into the clinical areas and the settings where task-shifting has been adopted, with important lessons for policy and practice. Since many studies employed non-randomized study designs, often with poor comparability between intervention and control arms, most articles in this review were deemed to be at moderate or serious risk of bias. Many studies failed to account for patient, procedure and healthcare setting characteristics, leading to imbalances between patient groups. For instance, specialists may have managed patients at higher risk of experiencing poorer outcomes and performed the most complex operations. It is also possible that non-specialists may have been more likely to be stationed in rural and public hospitals, which are often underresourced and lack accessibility to life-saving equipment or appropriate referral systems.

It was not uncommon for retrospective studies to report high rates of missing outcome data caused by the difficulty in retrieving patient files and their incompleteness. In studies where non-specialists operated under supervision, the direct involvement of physicians in the procedures often remained unclear, possibly leading to biased results. Finally, many studies included a short follow-up period, often measuring perioperative or in-hospital mortality and morbidity rates. However, anaesthetic and surgical complications are likely to manifest over time. Our review has also highlighted that there is a substantial gap in the literature responding to the research question beyond sub-Saharan Africa and the USA. This is not surprising, as surgical task-shifting is illegal in many parts of the world, but it is common practice in many countries across Africa.

### Implications for future research

Further research is needed to understand the implications of surgical task-shifting in middle-income countries and HICs and to better assess the performance of non-specialist anaesthesia providers, given the scarcity of available literature. Studies on surgical task-shifting should adopt a broader lens, performing a robust analysis of all aspects of perioperative care, beyond non-specialists’ technical performance in the operating theatre. It remains essential to evaluate the care provided by non-specialists across the perioperative pathway, including preoperative and postoperative management. Focus must also be placed on non-technical skills, such as clinical decision-making, communication and situational awareness. To strengthen the quality of the available evidence, future studies should adopt randomized study designs and include long-term outcome measures to generate high-quality evidence.

### Policy implications

Our results suggest that task-shifting in low-complexity procedures such as caesarean sections and hernia repairs may be implemented safely. This is an important finding, given the high unmet need in obstetrics and general surgery in many low-resource settings (Bolkan *et al.*, 2016; Lindheim-Minde *et al.*, 2021). However, the effective adoption of task-shifting requires a modification of existing regulatory and governance systems, with formal recognition by Ministries of Health and professional bodies within medical practice regulations and national workforce strategies (Baine and Kasangaki, 2014; Ashengo *et al.*, 2017; Jumbam *et al.*, 2022). Evidence-based guidelines are required to define the precise scope of practice of non-physicians and non-specialist physicians and to establish credentialed pre-serving training curricula, ensuring that clinicians can operate safely and within a well-defined legal framework. This may include the incorporation of task-shifting as a workforce development strategy to address surgical need into National, Surgery, Obstetrics and Anaesthesia Plans in healthcare systems across low-, middle- and high-income country settings. Anaesthetic and surgical task-shifting policies must also be accompanied by systems of prospective recording and reporting, as well as the development of regulatory systems for quality assurance. It is essential to rigorously monitor long-term outcomes across all countries where task-shifting is implemented to evaluate the performance of lower-level cadres and to ensure accountability (Schroeder *et al.*, 2020).

Training programmes should be recognized as an indispensable component of successful task-shifting initiatives. These enable the extended surgical team to develop technical skills as well as non-technical skills, including decision-making, effective communication and situational awareness. Importantly, training ought to be tailored to local contexts, preparing trainees for the challenging working conditions, which they would face in poorly resourced settings (Edgcombe *et al.*, 2018; Kudsk-Iversen *et al.*, 2018; Igaga *et al.*, 2021). After qualification, supervision, inter-professional collaboration and continuing education are integral to enable anaesthesia and surgical providers to maintain and strengthen their competencies over time (Edgcombe *et al.*, 2018; Kudsk-Iversen *et al.*, 2018). To guarantee sustainability, one option could be to investigate how digital telecommunication technologies and e-learning platforms can be leveraged in resource-constrained settings to better connect specialists and non-specialist providers and facilitate access to professional development opportunities (Drum and Bould, 2017; Igaga *et al.*, 2021; Mwapasa *et al.*, 2021). Task-shifting cannot substitute specialist physicians, who remain integral to performing complex procedures, making informed clinical decisions and training surgical care providers (Beard *et al.*, 2014; Kudsk-Iversen *et al.*, 2018). In line with an inter-professional approach, task-shifting must be implemented alongside investments in ‘standard’ speciality training programmes for anaesthesiologists and surgeons. Both policy approaches are not mutually exclusive and should be developed contemporarily and complementarily to strengthen anaesthesia and surgical delivery globally.

Although task-shifting has been extensively advocated as a solution for the human resources crisis in global surgery, some studies have suggested that trained non-specialists may also be prone to leave the areas in greatest need to seek personal opportunities elsewhere (Schneeberger and Mathai, 2015; Gajewski *et al.*, 2019a; Asefa *et al.*, 2020). For example, Asefa *et al.* (2020) reported attrition rates as high as 65.9% and 71.4% for non-specialist surgical and anaesthetic personnel 15 months after deployment to rural hospitals. It remains crucial to research incentives to upkeep healthcare workers’ motivation and facilitate their retention. Financial measures to attract non-specialist staff might include higher salaries coupled with bonding schemes, performance-based financing and hardship allowances together with social amenities, such as housing subsidies, health insurance and well-performing schools for their families. Furthermore, recognition, programmes for further training and specialization, clear career pathways and opportunities for professional growth appear to be indispensable to favour job satisfaction (Baine and Kasangaki, 2014; Nyawira *et al.*, 2022).

## Conclusion

Research predominately from sub-Saharan Africa suggests that non-specialists have performed common and low-complexity procedures, including caesarean sections and general surgery, with comparable outcomes to specialist physicians, thereby increasing access to safe and timely essential surgery. However, there remains a lack of high-quality research on the safety of surgical task-shifting for more complex procedures in LMICs and to further study the adoption of this strategy in HICs. More focus must be placed on anaesthesia care to better assess the performance of non-specialist providers in this field. In all contexts, for task-shifting to be sustainable and effective, policy efforts must centre around its formal recognition in regulatory and governance systems, around strengthening adequate training and supervision programmes and investigating strategies to favour the retention of healthcare workers in resource-poor contexts.

## Supplementary Material

czad059_Supp

## Data Availability

All available data will be shared upon request.

## Reflexivity statement

The research team was inclusive, presenting a balance of female and male authors, seniority levels and clinical and non-clinical experience.

## Registration

PROSPERO registration number: CRD42022303527.
