# Supplementary material for: Assessing the impact of anaesthetic and surgical task-shifting globally: a systematic literature review
Source: Health Policy Plan. 2023 Jul 28;38(8):960–94. doi: 10.1093/heapol/czad059 (PMC10506531; doi:10.1093/heapol/czad059)
Supplement: czad059_Supp [file czad059_supp.zip › suppl_data/10. Health Policy and Planning, Supplementary file 1. .docx]

**Supplementary File 1**

**I. MEDLINE search strategy**

- Exp surgical procedures, operative/ or exp surgeons/ or (surger* or surgical* or surgeon*).tw
- Exp anesthetists/ or (anaesthesia or anesthesia or anaesthesiology or anesthesiology or anaesthetist* or anesthetist* or anaesthesiologist* or anesthesiologist*).tw
- (Cesarean section* or caesarean section* or c-section* or obstetric* or laparotom* or open fracture*).tw.
- Delegation, professional/ or (Task* adj3 (shift* or share* or sharing* or substitut* or delegat*)).tw
- ((Substitut* or delegat* or shortage* or shortfall*) adj3 (physician* or doctor* or surgeon* or anaesthetist* or anesthetist* or anaesthesiologist* or anesthesiologist* or specialist* or professional* or surgical workforce or surgery workforce or anaesthesia workforce or anesthesia workforce or health workforce or health care workforce or healthcare workforce or health personnel or trained personnel or health worker* or health care worker* or healthcare worker* or health provider* or health care provider* or healthcare provider*)).tw
- (Non-surgeon* or nonsurgeon* or non-physician* or nonphysician* or non-doctor* or nondoctor*).tw
- (Non-specialist* physician* or nonspecialist* physician*).tw
- (Physician assistant*).tw
- (Physician extender*).tw
- (Medical officer* or clinical officer*).tw
- ((Midlevel or mid-level) adj2 (provider* or practitioner*)).tw
- (Medical technician*).tw
- (Associate clinician*).tw
- ((Surgeon* or surgical) adj2 (associate or associates or assistant* or technician* or practitioner* or officer*)).tw.
- ((Anaesthesia or anesthesia or anaesthetic* or anesthetic*) adj2 (associate or associates or assistant* or technician* or practitioner* or officer*)).tw
- **(**Certified registered nurse anesthetist* or certified registered nurse anaesthetist*).tw
- (Advanced practice provider*).tw

**Limits for MEDLINE:**

- Year range: 2008-Current
- Letter/ or editorial/ or news/ or exp historical article/ or anecdotes as topic/ or comment/ or (comment or editorial or letter).pt
- (Exp animals/ or exp animals, laboratory/ or exp animal experimentation/ or exp models, animal/ or exp rodentia/ or (rat or rats or mouse or mice).ti) not humans/

**II. ROB-2. Risk of bias judgements and reasonings for randomised studies.**

**III. ROBINS-I. Risk of bias judgements and reasonings for non-randomised studies.**
